# Effect of Summer Holiday Programs on Children’s Mental Health and Well-Being: Systematic Review and Meta-Analysis

**DOI:** 10.3390/children11080887

**Published:** 2024-07-23

**Authors:** Emily Eglitis, Catherine Simpson, Ben Singh, Timothy Olds, Amanda Machell, Rosa Virgara, Mandy Richardson, Kylie Brannelly, Aniella Grant, Jessica Gray, Terri Wilkinson, Zoe Rix, Carol Maher

**Affiliations:** 1 Alliance for Research in Exercise, Nutrition and Activity (ARENA), Allied Health and Human Performance, University of South Australia, Adelaide, SA 5000, Australiaben.singh@unisa.edu.au (B.S.); timothy.olds@unisa.edu.au (T.O.); amanda.watson@unisa.edu.au (A.M.); rosa.virgara@unisa.edu.au (R.V.); graam012@mymail.unisa.edu.au (A.G.); grajy036@mymail.unisa.edu.au (J.G.); wiltj031@mymail.unisa.edu.au (T.W.); rixzd001@mymail.unisa.edu.au (Z.R.); carol.maher@unisa.edu.au (C.M.); 2 Centre for Adolescent Health, Murdoch Children’s Research Institute, Parkville, VIC 3052, Australia; 3 Department for Education, Government of South Australia, Adelaide, SA 5000, Australia; mandy.richardson@sa.gov.au; 4 National Outside School Hours Services Alliance (NOSHA), Woodend, QLD 4305, Australia; kylie@qcan.org.au

**Keywords:** summer camps, children, mental health, anxiety, depression, distress, well-being, cognition

## Abstract

Poor youth mental health is an area of global concern. Summer holiday programs may provide environments that support mental health when the structures and supports of school are not available. The aim of this review was to determine the effectiveness of summer holiday programs in improving the mental health, social–emotional well-being, and cognitive (non-academic) outcomes of children and adolescents. Studies of summer holiday programs for school-aged children (5–18 years) were included if they measured any mental, socio-emotional or cognitive (non-academic) outcome. Studies were excluded if they were published prior to 2000, targeted clinical populations or lasted less than five days. Six databases were searched (April 2023). Risk of bias was assessed using the PEDro tool. Study outcomes were grouped according to three main constructs: mental health (psychological well-being, anxiety, depression, distress, and self-perception including self-esteem, self-worth, self-concept, confidence, and competence); social–emotional well-being (behavior and social skills, e.g., communication, bullying, conflict resolution, empathy, and social skills); and cognitive function (memory, selective attention, and executive function). A fourth “other” group captured substance use, personality traits, character skills, and values. Effect sizes were calculated as the standardized mean difference between pre- and post-intervention scores. The synthesis involved a random-effects meta-analysis (presented in forest plots), where possible, with the remaining outcomes narratively synthesized. Twenty-six studies (n = 6812 participants) were included. The results of the meta-analysis suggested that summer programs showed a statistically non-significant trend toward reducing symptoms of anxiety and depression (k = 2 studies, SMD = −0.17, 95% CI −2.94, 2.60), psychological distress (k = 2 studies, SMD −0.46, 95% CI –1.71, 0.79), and no effect on self-esteem (k = 6 studies, SMD = 0.02, 95% CI −0.02, 0.06) or self-worth (k = 3 studies, SMD = 0.05, 95% CI 0.00, 0.11). Narrative syntheses indicated a pattern toward improvements in general mental health, self-perception, social–emotional outcomes, and cognition. Studies were generally small, with a high risk of bias. Summer holiday programs for children and adolescents show trends toward improving mental, social, emotional, and cognitive outcomes. Programs targeting disadvantaged children showed stronger patterns of improvement related to mental health and self-perception than programs targeting the general population. While effect sizes are small to negligible, they consistently indicate improvements. Summer programs present a promising avenue to promote mental health in children; however, further rigorously designed, clearly reported control-group studies are required to more fully understand their effects.

## 1. Background

A healthy childhood lays the foundation for lifelong health. Mental health, as defined by the World Health Organization, encompasses a state of well-being in which individuals recognize their abilities, can manage normal life stresses, work productively, and contribute to their community [1]. The dual-factor model of mental health suggests that true mental health includes both the absence of mental illness (e.g., symptoms or disorders) and the presence of positive psychological well-being and emphasizes the importance of addressing both negative symptoms and fostering positive aspects such as life satisfaction, happiness, and personal growth [2,3]. Mental health is an important aspect of overall health, and it is critical to healthy social and emotional development, and problem-solving and coping skills [4]. Positive mental health allows children to more fully realize their academic potential [5]. Conversely, mental health disorders such as anxiety and depression reduce children’s quality of life across domains of psychological, physical, and social well-being [6,7].

Poor mental health of children and adolescents is a serious area of concern globally, as depression, anxiety, and behavioral disorders are the leading cause of illness and disability in 10–19-year-olds [8]. The symptoms of poor mental health are not always clearly visible. They may manifest internally, such as through anxiety, depression, and psychological distress; or externally, such as through aggression and hyperactivity. Research indicates that internalizing symptoms are generally more prevalent in children and adolescents than externalizing symptoms [9]. Compared to children with good mental health, children that suffer a mental health condition are six times more likely to suffer a mental health condition in adulthood [10] and go on to earn significantly lower income [11].

Environmental factors play an important role in the mental health of young people [12], and children spend a large proportion of their time in the school environment. School supports the development of positive mental health through consistent engagement in educational activities that positively impact students’ cognitive development [13,14,15]. Participation in classroom learning also requires children to practice executive functions, including memory, selective attention, and inhibitory control [16]. Engagement in enjoyable school-based and extra-curricular activities can be rewarding in and of itself, providing opportunities to learn new skills and achieve mastery in meaningful areas, thus increasing self-worth. Meanwhile, tailored strategies can help enhance a more stable, intrinsic sense of self- acceptance, which is referred to as self-esteem [17]. The social interaction and structured routines of the school day also help children develop social and emotional skills. Teachers can further provide emotional support and set clear expectations and boundaries that help children feel safe, ease anxiety, and develop a sense of belonging [18]. Teachers can also identify students struggling with the social and cognitive demands of schooling, thus allowing for early intervention. School provides opportunities to meet peers and form friendships, thus playing an important role in social development [4,19] that can protect against social maladjustment [20]. Furthermore, schools help children to display more favorable behavior patterns, including more time in physical activity and less time engaged in sedentary behaviors, like recreational screen-use [21,22]. This can positively support children’s mental health [23,24]. For example, decreasing screen use helps to improve sleep habits, supporting, in turn, good mental health [12], while increasing physical activity in children has been shown to reduce anxiety and depression and improve self-esteem and cognitive function [25].

While school holidays offer a reprieve to students and families from the demands of the school term, extended periods of time spent away from the school setting reduces the supports provided by school, the absence of which may negatively impact children’s mental health. Some evidence suggests that negative experiences over the summer holidays (like loneliness or food insecurity) can increase anxiety and negatively influence children’s mental health, with disadvantaged children affected to a greater extent [26,27]. Negative peer-group interactions over summer have been linked with increases in anti-social behaviors (bullying and victimization) [28], while reduced peer contact can reduce children’s enjoyment of, and therefore time spent in, physical activity [29].

Summer programs have proven benefits in a range of other outcomes for children, and it is feasible that some of the beneficial features of summer programs also enhance mental, emotional, and social well-being. Previous reviews of summer programs looking at academic outcomes found improvements in mathematics and reading achievement [30,31,32], while reviews of health outcomes have found moderate reductions in sedentary behavior and small increases in moderate-to-vigorous physical activity [33,34]. To date, no reviews have synthesized the evidence regarding the impact of summer programs on social, emotional, cognitive, and psychological outcomes. Thus, the objective of this systematic review was to determine the effectiveness of summer holiday programs in improving the mental health, social–emotional well-being, and cognitive (non-academic) outcomes of children and adolescents. The review questions were as follows:

1. What are the effects of summer holiday programs on children and adolescents’ mental health, well-being, and social–emotional and cognitive function?
2. Do the effects differ based on child characteristics (e.g., socioeconomic disadvantage and age)?
3. Do the effects differ based on program characteristics (e.g., program content and duration)?

## 2. Methods

### 2.1. Protocol and Registration

A systematic review protocol was registered prospectively with PROSPERO (registration number: CRD42023409799) [35] and reported following the PRISMA 2020 guidelines [36].

### 2.2. Eligibility Criteria

Participants in the included studies were school-aged children (5–18 years) participating in a summer program (intervention) of at least five days’ duration that was exclusively delivered during the summer period (i.e., not including afterschool or other holiday programs). Primary outcomes included any mental health (e.g., psychological well-being, depression, and anxiety), socio-emotional (e.g., social connectedness, sadness, loneliness, appropriate behavior), or cognitive (e.g., working memory and executive function) outcome. Academic outcomes were not included, because they have been the focus of other reviews [30,31]. Studies were excluded if they were published before 2000 or if they targeted clinical populations (e.g., cancer and diabetes), special needs (learning or intellectual disabilities), or gifted/especially talented children. Study designs included both experimental studies with a control group, receiving “summer as usual” or a different summer program, and quasi-experimental (pre-post-intervention measures with no control group). No language limits were set. Full criteria are given in Supplementary File S1.

### 2.3. Information Sources and Search Strategy

Six databases were searched for peer-reviewed original articles: Embase, MEDLINE, JBI, PsychINFO (via OVID), ERIC, and Scopus (April 2023). A broad search strategy [37] was developed with an academic librarian, focusing on population and context terms (detailed in Supplementary File S2). Reference lists of included studies were searched using Citationchaser (version 0.0.3, Haddaway, Grainger and Gray 2021), and the corresponding authors of included studies were contacted to identify further relevant studies. 

### 2.4. Selection Process

Search results were imported into Endnote 20 (Clarivate Analytics, Philadelphia, PA, USA), where duplicates were removed before being imported into ASReview [38] (version 1.1.1, ASReview LAB developers, Utrecht, The Netherlands), where the five same relevant studies identified during preliminary searches were used to train the search. Two independent reviewers (EE and BS) completed title and abstract screening, which was stopped once 10% of the total studies were screened and 100 consecutive, irrelevant titles were encountered. Full-text review was then completed using Covidence (Veritas Health Innovation, Melbourne, Australia). Screening was completed by EE and a second independent reviewer (TW or ZR), and disagreements were resolved through discussion. 

### 2.5. Data Collection Process and Data Items

Charting tables were developed by the authorship team and piloted prior to use (example in Supplementary File S3). Fields for data extraction included study features (design, geographical location, and participant number); participant demographics (age, gender, and socioeconomic status); program design (structure, objectives, and environment); findings (measures of effect, certainty, and statistical significance, measurement tool, and timing of evaluation); and implementation outcomes (adverse events and attendance). Data extraction and risk-of-bias assessment were completed in duplicate using Covidence by two independent reviewers (EE, TW, ZW, JG, CS, and AG), with discrepancies resolved through discussion until consensus was reached.

### 2.6. Study Risk-of-Bias Assessment

Risk-of-bias assessment was completed in duplicate using Covidence by two independent reviewers (EE, TW, ZW, JG, CS, and AG), by employing the PEDro Risk of Bias Tool, recognized for its validity and reliability [39]. The highest possible PEDro score of 10 signifies minimal risk of bias, with categories used to assign studies as low, moderate, and high risk of bias [39]. However, the impracticality of blinding people delivering the program meant that this item was omitted, and the maximum score was revised to nine [40]. The interpretation of scores in this paper was 7–9 (low risk of bias), 5–6 (moderate risk), and 0–4 (high risk of bias). 

### 2.7. Effect Measures and Synthesis Methods

Studies were grouped with the assistance of a behavioral scientist according to three main constructs: mental health, social–emotional well-being, and cognition. Mental health included psychological well-being (e.g., anxiety, depression, distress, well-being) and self-perception (e.g., self-esteem, self-worth, self-concept, confidence, and competence). Social–emotional well-being covered behavior and social skills (e.g., communication, bullying, conflict resolution, empathy, and social skills), while cognitive function included outcomes related to memory, selective attention, and executive function. Outcomes falling outside of these main constructs were presented separately as “other” and included health-related behaviors (substance use), personality traits/character skills, and spirituality/values. Effect sizes were calculated as the standardized mean difference from pre- and post-intervention scores and were interpreted as small (0.2), moderate (0.5), or large (0.8) [41]. Synthesis involved meta-analysis where possible, with the remaining outcomes narratively synthesized. Patterns were examined for each outcome when at least three outcomes were available. 

### 2.8. Meta-Analysis

Meta-analyses were conducted for outcomes with sufficiently homogeneous data from at least two studies. Study authors were contacted for missing data. A random-effects model was used for meta-analyses, considering heterogeneity in study design and outcomes reported. Meta-analyses were conducted using R software (version 4.3.1) with the meta, metafor, and dmetar packages [42,43,44,45,46]. Standardized mean differences and associated confidence intervals were used to indicate the size and precision of the effect estimate, with restricted maximum-likelihood and Knapp–Hartung adjustments [46]. Meta-analysis results were presented using forest plots. Interpretation of results considered the direction, size, and precision of the effect with statistical significance set at *p* = 0.05. Patterns across studies were also considered. The *I*^2^ statistic was used to evaluate statistical heterogeneity, with *I*^2^ > 50% indicating substantial heterogeneity [41]. Robustness of results (effect size, statistical significance, and heterogeneity) was explored via a sensitivity analysis, using leave-one-out analyses, and analyses were repeated, excluding studies possessing a high risk of bias. Publication-bias analysis was omitted due to the small study set [47]. 

### 2.9. Narrative Synthesis

Where meta-analysis was not possible, a narrative synthesis was conducted according to the synthesis without meta-analysis (SWiM) guidelines [48]. Standardized measures of effect (e.g., odds ratio; mean difference; and standardized mean difference with statistical significance determined by the original study, usually *p* = 0.05) were used to code outcomes as improved, unchanged, or declined. To indicate the magnitude of intervention effect, a common effect metric was used: SMD (Cohen’s D, Hedges’ g) was prioritized, and when not available, it was calculated from baseline and post-intervention scores or converted from other effect metrics, such as eta-squared [49]. 

### 2.10. Certainty of Evidence

The overall evidence for each outcome was graded based on the Oxford Centre for Evidence Based Medicine’s (OCEBM) 2011 Levels of Evidence [50,51]. This approach evaluates an intervention’s effectiveness using a hierarchy of evidence, considering study design, effect size, and consistency of effects across studies. First, studies’ designs and effects were rated on a scale from one (highest) to five (lowest). Then, the evidence for each outcome was graded A–D: Grade A was assigned to outcomes with consistent level 1 studies (randomized controlled trials); Grade B for consistent level 2 studies (non-randomized controlled studies); Grade C for consistent level 3 studies (non-randomized, controlled studies, and for this review, repeat-measure non-controlled studies); and Grade D for inconsistencies in the direction of effect. Grades were adjusted down for high risk of bias and inconsistency between studies and adjusted up for large and consistent effect sizes. A minus sign indicated considerable clinical or statistical heterogeneity (*I*^2^ ≥ 50%).

### 2.11. Deviation from Registered Protocol

There were limited numbers of experimental trials with control groups; therefore, the original inclusion criterion of only experimental controlled study designs was broadened to include quasi-experimental designs (e.g., pre–post-intervention measures with participants acting as their own controls). Subgroup analyses based on child (age and SES) or program (duration and format) characteristics were undertaken narratively. 

## 3. Results

### 3.1. Study Selection

**Figure 1:**
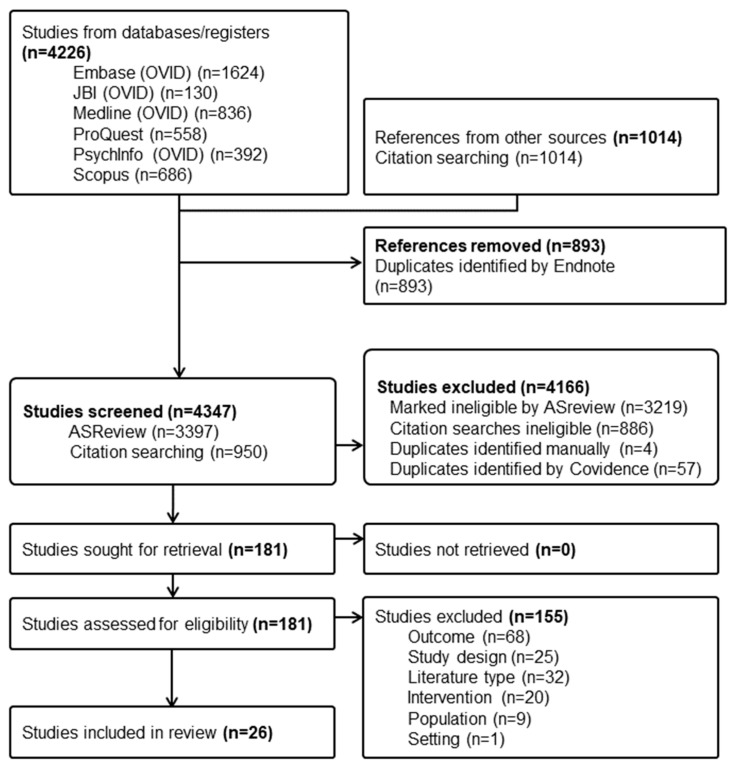
PRISMA flow diagram of study identification, screening, and inclusion.

Database searching yielded 4226 studies, with reference-list searching adding 1014 more. After the removal of duplicates, 4347 titles and abstracts were screened with 181 full texts retrieved. Finally, 26 studies involving 6812 participants were included (Figure 1). A full list of studies excluded at the full-text screening stage and their reason for exclusion is presented in Supplementary File S4.

### 3.2. Study Characteristics

Included studies are summarized in Table 1. A summary of program and participant characteristics is presented in Table 2 (thematic coding is described in Supplementary File S5). Three studies were randomized controlled trials (RCTs) (k = 2 cluster RCTs [52,53], k = 1 individual RCT) [54], three were non-randomized controlled trials [55,56,57], and the remaining studies were within-subject, repeated-measures of groups all receiving an intervention (k = 2 studies had three groups [58,59], k = 1 study had two groups [60], and k = 17 studies had a single intervention group [61,62,63,64,65,66,67,68,69,70,71,72,73,74,75,76]). Sixty-two percent of the studies were from the United States (k = 16), with two studies from India and single studies from Australia, Canada, Italy, Japan, Spain, Switzerland, Turkey, and the United Kingdom. Ten of the eleven interventions that targeted children disadvantaged by low family SES or race were conducted in the US [52,59,63,68,69,70,71,74,75], with the remaining study conducted in Spain [64]. In 42% of studies (11/26 studies), program attendance was funded on behalf of the participants (e.g., trial funding, community organization, and scholarships) [52,53,54,58,62,64,68,69,71,73,75]; in two cases, the participants paid in full [55,72], and in another three studies, participants paid part of the fees with scholarships or third-party funding paying the gap [67,70,76]. Only 38% of studies (10/26) reported attendance levels, and most of these (k = 7) reported high attendance (categorized as ≥66% of the sessions) [54,63,65,69,70,71,73]. One study reported moderate attendance (51–65% of the sessions) [67], one reported low attendance (≤50% of the sessions) [77], and another reported different low-to-high attendance levels across three different sites [52]. One study reported no adverse events [73], while all other studies did not mention adverse events. Thus, no studies described experiencing adverse events.

A diverse range of outcomes were reported across the studies, and a brief definition of each broad construct is provided here for readers unfamiliar with this literature. First, “mental health” is used here to describe critical components of psychological well-being, encompassing symptoms of anxiety, depression, and psychological distress, as well as items related to self-perception, including self-esteem (the overall sense of value or worth) and self-worth (value derived from specific achievements or attributes) [78,79,80]. “Social–emotional” outcomes relate to one’s ability to comprehend and regulate emotions, form and sustain healthy relationships, and effectively interact within social contexts. This includes key social skills, such as communication, cooperation, assertion, problem-solving, responsibility, and self-control [81]. “Cognitive” outcomes measure mental processes involved in gaining knowledge and understanding, and they encompass a range of cognitive functions, including memory, attention, executive function, and decision-making [82].

### 3.3. Program Characteristics

Most interventions were day camps (k = 15) [52,54,56,59,61,62,63,65,66,67,68,69,71,74,75], five were residential (overnight) camps [55,58,70,72,76], and one studied both day-camp and residential formats [73]. Two were delivered at home [53,77]. School was the most common setting for interventions (k = 10 [52,54,55,59,61,62,63,67,74,75]), followed by private organizations (k = 4 studies), including yoga training centers [60], outdoor recreational settings [70], or summer camp [76] facilities or horse-riding schools [72]. Two studies included a diverse range of camps run by different providers [56,73], and one was run by a church-based community group [58]. Partnerships were sometimes formed to share settings, resources, and facilities across sites between schools and community-based organizations, like homeless shelters [68] or local councils partnering with a local urban farm business [69]. Five studies did not specify the setting [57,64,65,66,71].

Just over a third of interventions (k = 10) ran between one and two weeks from start to finish [54,57,58,60,64,65,66,70,72,76]. Single studies lasted for three [71], four [61], or five weeks [75] each. Five studies lasted for six weeks [62,63,67,69,74]. Four studies had ranges of duration (either by allowing participants to select how many sessions they attended or using a research design that contrasted shorter- vs. longer-duration interventions). For these studies, the minimum duration started at 1–5 weeks and went to a maximum of 4–6 weeks [55,59,68,73]. The longest intervention was eight weeks [52]. Three studies did not report the duration of interventions [53,56,77].

Regarding attendance, all but the two home-based programs [53,77] involved daily attendance of 4–5 days per week. Regarding contact hours, ten studies conducted programs using full-day schedules (≥7 h) [55,58,60,63,65,69,70,72,74,76], while four studies each ran their programs during the usual school hours [52,67,68,71] or a half-day schedule [54,61,66,75]. Two home-based programs [53,77] conducted intermittent group sessions, and two others conducted daily sessions lasting approximately two hours (one focusing on building specific conflict resolution skills [62] and another delivering counselling within an existing camp program [59]). 

The content of interventions was primarily based around general enrichment (recreation, sports, and play-based programs), with some also targeted toward addressing specific mental health and well-being factors (e.g., overall mental health [53,68,74], stress reduction [59], development of social skills [62,64,77], or reduction in risk behaviors [53,54,66]). Some programs solely targeted physical health [55,75] (e.g., physical activity and weight control), while others combined physical and mental health components [52,76]. Two broad studies covering multiple sites and programs did not report the program content [56,73]. 

### 3.4. Participant Characteristics

Studies were categorized into school levels based on the age range of their participants. Primary-school children (kindergarten/reception to grade six, aged from five to 11 years) were targeted in ten studies [52,62,63,65,66,67,68,70,73,77]. Six studies focused on middle-school students (grades 7–9, aged 12–14) [53,58,60,64,72,76]. Three studies focused on high-school aged students (grades 10–12, ages 15 and older) [54,59,69], and seven studies included children across all three age categories [55,57,61,71,74,75].

## 4. Meta-Analysis

**Figure 2:**
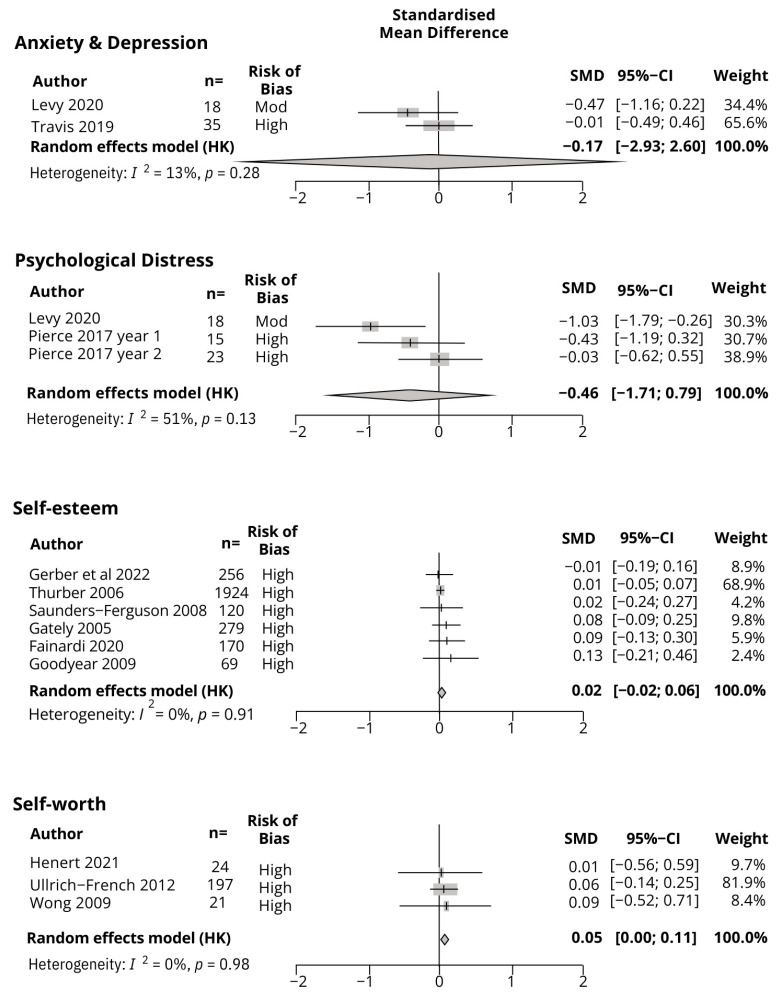
Meta-analysis results for anxiety and depression, distress, and self-esteem and self-worth.

Figure 2 presents the meta-analysis results for each construct, and Supplementary File S6 provides the full results, along with sensitivity analyses.

### 4.1. Mental Health

A meta-analysis was conducted on combined anxiety and depression scores (n = 53) [59,74] (one study reported only the aggregate anxiety and depression scores [74]; thus, anxiety and depression scores had to be combined). The result indicated that summer programs had a small but statistically non-significant effect in reducing symptoms of anxiety and depression (SMD = −0.17, 95% CI −2.94, 2.60, *p* = 0.0.58, I^2^ = 13%). Both studies showed a trend toward improvement. Certainty of evidence: Grade C.

A meta-analysis was conducted on psychological distress in two studies (n = 56 participants) [59,69]. The result indicated that summer programs had a small/moderate but statistically non-significant effect in reducing symptoms of psychological distress (SMD −0.46, 95%CI –1.71, 0.79, *p* = 0.26, *I*^2^ = 51%). A sensitivity analysis identified Levy et al. (2020) [59] as an influential case, and when omitted from the analysis, the effect size reduced to small and remained statistically non-significant. All studies showed a trend toward an improvement. Certainty of evidence: Grade C.

A narrative synthesis was conducted on mental health data from four studies (n = 714) [52,54,58,65]. Two studies measured general mental health and found either a small improvement [65] or no change [54]. Two measured a positive/negative affect: one found improvements [52], and the other (which investigated various settings, curricula, and facilitation strategies across three different intervention programs) also measured life satisfaction and found mixed results for all outcomes [58]. Overall, there was a trend toward improvements in general mental health. Certainty of evidence: Grade C-.

### 4.2. Self-Perception

A meta-analysis was conducted for self-esteem, based on six studies (total n = 2818) [55,56,65,66,72,73]. The results indicated that summer programs had no effect on self-esteem (SMD = 0.02, 95%CI −0.02, 0.06, *p* = 0.21, *I*^2^ = 0%). Five of the six studies showed a trend toward improvement; however, effect sizes were negligible. The effect of summer programs on self-esteem is negligible. Certainty of evidence: Grade C.

A meta-analysis was conducted for self-worth, based on three studies (n = 424 participants) [67,75,76]. The results indicated that summer programs had no effect on self-worth (SMD = 0.05, 95%CI 0.00, 0.11, *p* = 0.05, *I*^2^ = 0%). The direction of effect was consistent across studies; however, the effect sizes were negligible. Overall, the effect of summer programs on self-worth is negligible. Certainty of evidence: Grade C.

A narrative synthesis was conducted on self-perception-related outcome measures from nine studies [61,63,67,70,72,73,74,75,76]. These included self-concept, self-perception, self-efficacy (competence and confidence), physical appearance, and identity. For self-concept, three studies found improvements [70,76], with one also finding improvements in racial identity [63]. Two found improvements in general, sport, or social competence [61,74], and two more studies found improvements in self-perceptions [73,75]. Two studies found no change in physical appearance and self-acceptance [67,72]. Overall, there was a general trend toward improvements in self-perception. Certainty of evidence: Grade C-. 

### 4.3. Social–Emotional Well-Being

A narrative synthesis was conducted on social–emotional outcomes from fourteen studies (n = 4771 participants) [54,58,61,62,63,64,66,67,68,71,73,74,76,77]. For emotional and behavioral outcomes, an improvement was found in self-control [71], but no change was found in externalizing behaviors [71] or behavioral conduct [76]. Mixed findings were also found when teachers simultaneously rated children’s behaviors as deteriorating while parents rated them as improved [68]. Regarding communication skills, a single study was available that was suggestive of an overall improvement [77]. For pro-social behaviors, improvements were seen in empathy [64], social skills [63,73], social acceptance [76], social competence [67], relationships and coping with problems [66], and aspects of conflict resolution [62]. Other studies found no effect on connection, caring [74], social competence, or belonging [61]. One study had mixed results for social skills across intervention groups that received different curriculum and facilitation strategies [58]. A final study looked at bullying and found improvements in victimization, but no change in perpetration [54]. There was an overall trend toward improvements in social–emotional well-being across the heterogeneous measures of social–emotional well-being. Certainty of evidence: Grade C-.

### 4.4. Cognition

A narrative synthesis was conducted on cognitive outcomes, including memory [57,60] and executive function [58] (n = 274). All three studies found improvements, with effects ranging from small to large. Certainty of evidence: Grade C.

### 4.5. Other Outcomes

The remaining outcomes included health-related behaviors (substance use, k = 2 RCTs), personal attributes, and character skills (k = 3 studies) and values (k = 2 studies). For substance use, findings were conflicting, with one finding a small improvement [53] and the other reporting worsening [54]. For personal attributes and character skills, one study each found improvements in physical and thinking skills (related to awareness and enjoyment of the environment) [73] and altruism [56], while two other studies found no changes in “horsemanship attributes” (which included responsibility and motivation) [72] or mindfulness [69]. Certainty of evidence: Grade D-.

## 5. Subgroup Analysis

Subgroup analyses were performed using count-based methods considering participant characteristics (disadvantage and age) and program features (duration, format, content, funding, and daily contact hours) for each outcome category. The results are detailed in Supplementary File S7. 

### 5.1. Participant Characteristics

A subgroup analysis based on disadvantage was conducted on all 26 studies. Studies that reported outcomes exclusively disadvantaged populations (i.e., low SES or racial minority, n = 18 outcomes) were compared to those from populations not identified as disadvantaged (i.e., mixed SES groups, SES/race not reported, n = 25 outcomes). Disadvantaged children showed more improvements in self-perception, with a trend toward improvements in mental health. Non-disadvantaged studies showed clearer improvements in social–emotional outcomes. A subgroup analysis based on participant age was conducted according to broad school categories (described previously, detailed in Supplementary File S5): primary school (n = 15 outcomes), middle school (n = 13 outcomes), and high school (n = 6 outcomes). There were no clear patterns based on children’s age.

### 5.2. Program Characteristics

A subgroup analysis was conducted based on format (residential vs. day programs), excluding studies where formats were mixed [73] or not reported [57,60,64]. Home-based programs [53,65,77] were also excluded due to the small number of heterogenous studies preventing meaningful grouping. Residential programs (n = 9 outcomes) targeted weight loss in obese children [55,76], programs for disadvantaged youth [58,70], and those attending a horse-riding camp. Day programs (n = 24 outcomes) [52,54,56,58,61,62,63,66,67,68,69,71,74,75] were much more varied. Day programs showed a clearer pattern of improvement for mental health and social–emotional outcomes. Self-perception improved across both formats.

A subgroup analysis was conducted based on content, comparing studies that had a curriculum with a specific mental, emotional, or social well-being content (n = 18 outcomes) [52,53,54,59,62,64,66,68,69,74,76,77] versus programs that did not (n = 25 outcomes) [55,57,58,60,61,63,65,67,70,72,73,74,75]. Specific programs showed stronger patterns of improvement in social–emotional well-being and self-perception, while non-specific curriculums favored mental health and cognitive improvements. 

A subgroup analysis was conducted based on daily contact hours: half day or less (n = 10 outcomes) [53,54,56,61,66,75,77] versus school day or longer programs (n = 24 outcomes) [52,55,58,59,62,63,65,67,68,69,70,71,72,74,76]. Social–emotional and self-perception outcomes improved across both groups, with clearer patterns of improvement in shorter day programs.

A subgroup analysis was conducted based on program duration, comparing programs lasting less than three weeks (n = 18 outcomes) [54,58,64,65,66,70,71,72,76] to programs that ran for three weeks or more (n = 14 outcomes) [52,61,62,67,68,69,74,75]. Studies that did not specify a duration [53,56,77] or that had a range of attendance durations that crossed this cut-off [55,59,73] were omitted from the analysis. There were no clear patterns when comparing shorter- vs. longer-duration programs. Only short programs studied cognitive outcomes, and all demonstrated improvements.

A subgroup analysis was conducted based on the funding model for children’s attendance. Studies where children’s attendance was funded in part or full by the child’s family (n = 8 outcomes) [55,67,70,72,76] was compared to programs where children’s attendance was externally funded (e.g., by the research trial, n = 18 outcomes) [52,53,54,58,62,64,69,73,75]. Family-funded programs appeared to have clearer social–emotional improvements than programs for which students did not pay.

## 6. Risk of Bias

A majority of studies (k = 22) lacked a comparison group and therefore were considered high risk of bias [52,54,55,56,57,58,59,60,61,62,63,64,65,66,67,68,69,70,71,72,73,77]. Three were deemed moderate [53,74,75], and one study had a low risk of bias [76]. The items of most concern involved the lack of concealed allocation to treatment arms (achieved by one study [53]) and blinding of the participants [53,76] or evaluators to which study arm the participant was in [74,76], achieved by just two studies each. Self-esteem and self-worth only included studies designated as high risk of bias. The small number of low- or moderate-risk-of-bias studies meant that sensitivity analyses based on risk of bias were not possible. The results for the PEDro risk-of-bias assessment are presented in Supplementary File S7. 

## 7. Discussion

This systematic review aimed to determine the effectiveness of summer holiday programs in improving the mental health, social–emotional well-being, and cognitive (non-academic) outcomes of children and adolescents. It included 26 trials, involving a total of 6812 children. The results revealed that previous studies have measured a wide variety of different mental, social, and cognitive outcomes, though the evidence base for any particular outcome is quite small. The results from the meta-analysis revealed that, while the directions of effects were generally favorable, effect sizes were small and not statistically significant for mental health outcomes (anxiety/depression and distress) and negligible for self-perception outcomes (self-esteem and self-worth). The results from the narrative synthesis indicated trends toward improvements in general measures of mental health, self-perception, social–emotional well-being, and cognition. Disadvantaged populations showed greater improvement in mental health and self-perception compared to non-disadvantaged populations.

First, we can explore how these programs compare to mental health-promotion programs in other settings. A review of systematic reviews (without meta-analysis) of mental health-promotion interventions for children (up to the age of nineteen) found that school-based interventions improved broad constructs of general mental health but did not change problematic behaviors, such as delinquency, conduct disorder, or school non-attendance [83]. More recently, a meta-analysis of school-based interventions that specifically targeted stress, anxiety, and depression found moderate (d = 0.62) improvements in depressive symptoms and no change in anxiety [84]. Targeted programs showed larger effects than universal programs. The effects found in our review are in the same direction as the reviews of school-based interventions, but they are smaller in size. This is most likely because summer program interventions are much shorter than school-based interventions, which can continue for a year or more [85]. Also, the studies included in our review were small and likely underpowered to reveal changes.

It is also important to consider that, in the context of the summer holidays, a lack of deterioration in outcomes may, in fact, indicate a positive finding. A vast majority of the included studies were pre- or post-intervention design, and while there were inconsistencies in the size of the effects across the outcomes, the directions of effects were almost never in the direction of deterioration. These findings should be overlaid on observational research that suggests that numerous outcomes decline during the summer holidays [86]; negative experiences over summer, like social isolation, hunger, and decreased physical activity, can have unfavorable consequences for children’s mental well-being, particularly for disadvantaged children [26]. Thus, a lack of decline may indicate that the summer programs actually did have a protective effect for children’s mental well-being. However, until there are high-quality studies with control group design, this uncertainty will remain unanswered.

The subgroup analysis based on participant characteristics suggested greater improvement in mental health and self-perception in disadvantaged populations compared to non-disadvantaged populations. This may be because children and adolescents from disadvantaged backgrounds are more likely to suffer from poor mental health [87] and, therefore, have more scope to improve over the course of the intervention than children already scoring well at baseline. This was the case for Thurber and colleagues [73] in their study of over five thousand children attending one of 80 accredited summer camps across the United States (n = 3395 completed both baseline and immediate post-camp measures). They found that children with the lowest scores pre-camp made the greatest gains over the course of the summer program. This could reflect a sharper contrast between the out-of-school environments typically experienced by disadvantaged children compared to non-disadvantaged children and, therefore, the greater relative benefits gained from being within the supportive networks and environments of summer programs.

The subgroup analysis based on program characteristics suggested a somewhat surprising pattern. While targeted programs appeared better at improving social–emotional outcomes than general programs, the reverse was the case for mental health and cognitive outcomes, where general programs seemed more effective. When we explored this finding further, we found that the targeted programs tended to recruit populations living with a greater number of risk factors for mental health issues [88], such as children living with mentally ill parents [66] or in homeless shelters [68], as well as disadvantaged populations (racial minority and low socioeconomic status). Taken together, our findings could indicate that social–emotional outcomes are more amenable to the interventions offered, while mental health outcomes are harder to change in the face of persistent structural barriers outside the scope of the summer program. Conversely, our findings may highlight the value of play and physical activity in enhancing youth mental health, as the general programs frequently focused on sports, outdoor recreation, yoga, and increasing physical activity.

It is important to acknowledge the study’s strengths and limitations. Importantly, this is the first review to explore the effect of summer programs on children’s mental, emotional, or social well-being. This review used rigorous systematic review methodology, including a highly comprehensive search strategy, and used a meta-analysis to synthesize data. It also considered a wide range of mental, emotional, and social outcomes. However, the review has a number of limitations, mostly related to the current state of the evidence base on this topic. The majority of included studies had small sample sizes and were rated as having a high risk of bias. Less than a quarter of the studies used a controlled design, which seems particularly prudent here, where previous observational studies have demonstrated clear time effects. While many outcomes were measured, there were inconsistencies in the use and reporting of validated outcome measures, with some studies using self-designed questionnaires. The majority of the studies were from the United States, limiting the generalizability to other contexts. While this limits our ability to draw strong conclusions from these data, there are still valuable insights to be gleaned on the role that summer holiday programs could play in improving the mental health of young people. 

There are several important implications from this review. First, it builds on our understanding of how summer programs can impact children’s health in a holistic sense. Summer programs have demonstrated effectiveness in improving academic outcomes including reading, writing [31], and mathematics [89], and physical health outcomes, including reducing sedentary behavior by increasing moderate-to-vigorous physical activity [55,90,91]. The current review highlights the quality and design issues of studies of summer programs targeting mental–emotional well-being. The same rigor that has been used to investigate health and academic outcomes should be adopted in future studies to explore the effect that summer programs have on mental and emotional well-being outcomes. Further research using rigorously designed and conducted RCTs is needed to test the effectiveness of specific mental health interventions delivered within (or as) summer holiday programs. Due to a lack of data on the mental health challenges children face and their “natural” trajectory over summer, control-group data will be vital to further help us understand the role that summer programs play in children’s overall mental health and well-being. Future intervention studies should clearly describe the components and structure of their interventions and use consistent, validated outcome measures to detect changes in mental, social, and emotional outcomes so that the most effective program structure and content can be identified. 

Summer programs may form part of a broader mental health-promotion strategy. Summer programs could complement school-based interventions by providing further small gains, or at least preventing regression, during a potentially high-risk time for declines. This could lead to cumulative effects when interventions in school and summer settings are run side by side [89]. But in order to improve mental health outcomes for entire populations, interventions need to be delivered at scale in real-world settings, a task that has proven exceptionally difficult thus far [85]. Taking an implementation science approach to future research is necessary and involves designing or adapting programs into potentially suitable large-scale delivery models. 

There are policy implications for these findings. Ensuring equitable access to mental health interventions is crucial so that the populations in need (e.g., disadvantaged families, those living with a greater number of risk factors for mental health disorders) are able to access them. Some have highlighted the need for greater cross-disciplinary collaboration in the design and implementation of trials, as such cross-disciplinary collaboration is currently hindered by existing policies and practices related to training, trial funding, and publishing [85]. Addressing these barriers requires concerted efforts to foster collaboration across various disciplines, ensuring comprehensive and inclusive mental health interventions.

## 8. Conclusions

The evidence base is affected by studies with a high risk of bias and clinical heterogeneity. Included studies generally show trends toward improvement, though often with small effect sizes. Disadvantaged children exhibited more significant improvements in self-perception and a trend toward better mental health. Future research on summer programs should incorporate validated measures of anxiety, depression, and psychological distress, utilizing high-quality, rigorously designed trials with clearly reported intervention components. Given the demonstrated improvements in academic and physical health outcomes, summer programs present a promising intervention strategy to mitigate health declines, especially in disadvantaged populations, during a potentially high-risk period.

## Figures and Tables

**Table 1:** Characteristics of included studies.

First Author (Year) Country	Study Design, Comparison	Participants Sample Size (n=, %F), Mean Age Years (SD)	Intervention Goal, Description, and Dose	Construct, Outcome (Outcome Measure) (OCEBM Level)
Anderson-Butcher [61] (2013) USA	Within subject, repeat measure (no control)	9–16-year-olds, primarily African American youth. n = 193 (43.5%F) Mean age: 11.9 (1.6)	Goal: Improve social competence and belonging, improve athletic competence Description: National Youth Sport Program. Focus on sport competence and enrichment: 2 h daily sport, social competence training (problem solving and assertiveness skills) and enrichment (inc. health/wellness activities, drug/alcohol prevention). Led by teachers, college students, members of community Dose: Day program (5 h/day), 5 days/w, 5 w duration	Social: Social well-being and self-competence: Social competence (4-item Perceived Social Competence Scale); Belonging (5-item Belonging Scale). Self-perception: Sport-specific social competence (Perceived Athletic Competence scale) *(3)*
Ay [62] (2019) Turkey	Within subject, repeat measure (no control)	Grade-4–6 children attending the Children’s University Summer School n = 44 (50%F) Mean age: not reported	Goal: Enhance conflict resolution, communication and anger management skills Description: Negotiation and Peer Medication training Program (understanding conflict and resolution, communication, anger management). Program implemented by classroom teachers with specific training Dose: Day program (1 h/day), 6 w duration	Social: Conflict Resolution (Conflict Resolution Skills Scale, subscales: integrating, dominating, obliging, avoiding, and compromising), Problem Solving (problem-solving confidence, self-regulation, and approach-avoidance) (Problem Solving Inventory for Children) *(3)*
Bethea [63] (2012) USA	Within subject, repeat measure (no control)	Inner-city African American Youth aged 5–14 years from low-SES backgrounds. n = 79 (44%F) Mean age: 8.6 (2.3)	Goal: To develop social skills, self-esteem, and racial Identity Description: Oakland Freedom School provided a culturally relevant arts and enrichment program inc. arts, crafts, food, games, sports, reading, music, and field trips. Dose: Day program (weekdays), 6 w duration	Social: Social skills (Wally Child Social Problem-Solving Detective Game, Social Skills Rating Scale) Self-perception: Racial Identity (Preschool Racial Attitude Measure II, Adolescent Survey of Black Life), Self-Concept (Purdue Self Concept Scale for Primary Grade Children, Student Self Concept Survey). *(2)*
De los Pinos [64] (2020) Spain	Within subject, repeat measure (no control)	12–17-year-old children from low-SES households at risk of problematic social or criminal behaviors. n = 113 (41%F) Mean age: 13.8 (1.5)	Goal: Increase empathy, foster coexistence between adolescents, prevent inappropriate behaviors Description: Positive behavioral interventions in the context of an educational summer camp using social and emotional learning approach. Individual, small- and large-group sessions. Led by trained social science graduates. Dose: Format not reported, 2 w duration.	Social: Social skills: perspective taking, fantasy, empathetic concern, personal distress (Spanish version of the Interpersonal Reactivity Index) *(3)*
Exner-Cortens [54] (2020) Canada	RCT	Grade-9 and -10 high-school students. N = 222 (68.5%F) Mean age: 15.5 (0.61)	Goal: Promote positive mental health, healthy relationships and reduce harm (substance use, bullying) Description: Healthy Relationships Plus classroom-based program involving education on communication, substance abuse, mental health, emotion regulation and healthy relationships. Delivered by teachers with specific program training Dose: Day program (2 h/day, for 8 days). 2 w duration.	Mental health: Positive Mental Health (social, emotional and psychological well-being) (Mental Health Continuum–Short Form) Social: Bullying (experienced or perpetrated physical, verbal, social, or electronic bullying) (Bullying Evaluation and Strategies) Other: Substance Misuse (variables from the Youth Risk Behavioural Surveillance Survey) *(1)*
Fainardi [65] (2020) Italy	Within subject, repeat measure (no control)	7–13-year-old children. n = 354 (48%F) Mean age: not reported	Goal: Increase self-esteem and quality of life through increased physical activity Description: Physical activity using sport and recreational activities. Led by professional trainers Dose: Day program (8.25 h/day), 5 sessions/w for 2 w	Mental health: Health Related Quality of Life (physical well-being, emotional well-being, self-esteem, family relationships, friend relationships, everyday functioning) (KidKindl child) *(3)*
Fujieda [77] (2011) Japan	Within subject, repeat measure (no control)	Public elementary-school children in grades 3–6. n = 247 (48%F) Mean age: not reported.	Goal: Improve communication skills Description: Training to parents and children to improve communication (greeting and listening) and social skills Dose: Home-based program (1× lecture, 1× workshop, 1× poster)	Social: Parent-child communication questionnaire
Gately [55] (2005) United Kingdom	Non-randomized controlled trial	Overweight or obese children (9–18 years). n = 357 (%F not reported. Mean age: not reported	Goal: To provide a safe supportive environment where children could reduce body mass whilst having fun Description: Structured daily fun-based PA, moderate dietary restriction, and lifestyle education (food choices, behavior change, and bullying) Dose: Residential program (2–6 weeks), 4× 1 h lifestyle educational sessions	Self-perception: Self-esteem (self-perception profile for children)
Gerber [56] (2022) Switzerland	Non-randomized controlled trial	French-speaking Swiss schoolchildren aged 6–16 years. n = 256 (54%F) Mean age: 10.4(2.1)	Goal: To investigate the impact that general summer camps have on socio-emotional development Description: Various Swiss holiday camps Dose: Various formats and durations (not specified)	Self-perception: Self-esteem (Self-Assessment Questionnaire) Social: Altruism (adapted: Rushton’s Self Report Altruism Scale) Other: Temperament (emotionality, activity, sociability, and shyness) (French Emotionality Activity and Sociability Questionnaire) *(2)*
Goodyear [66] (2009) Australia	Within subject, repeat measure (no control group)	6–13-year-old children from metropolitan Melbourne living with parental mental illness n = 69 (71%F) Mean age: 9.3	Goal: Enhance resilience, reduce isolation and provide enrichment, provide respite, education, and establish connections with health professionals Description: CHAMPS program: Age-appropriate mental illnesses education, promotion of healthy coping strategies using a strengths-based approach. Dose: Day program (5 h/day), 4 days/w, 1 w duration	Self-perception: Self-esteem (Self-esteem scale) *(3)* Social: Problem-focused coping (Kids Problems, Kids Coping) *(2).* Connections within family (Kids Connections) *(3)*
Henert [67] (2021) USA	Within subject, repeat measure (no control group)	Urban-dwelling children aged 6–13 years from mixed ethnic backgrounds. n = 24 (54%F) Mean age: 10.1 (2.2)	Goal: Develop pro-social life skills and increase physical activity Description: Camp Play-A-Lot program. Curriculum based on leadership and responsibility skills (taught through sports and physical activities) plus enrichment (arts, crafts, science, games, and offsite field trips). Delivered by trained counsellors. Dose: Day program (6 h/day), weekdays for 6 w (total 29 days)	Self-perception: Social competence, physical appearance, global self-worth (Self-Perception Profile for Children) *(3)*
Hopkins [52] (2019) USA	Cluster RCT (2 experimental arms, 1 standard-care control)	K–grade-5 children from low-SES, racial minority backgrounds N = 86 (57%F) Mean age: 7.6 (SE 0.2)	Goal: Physical and mental health promotion Description: Camp NERF: multi-component program combining nutrition and physical activity (CATCH), and mental health (COPE) interventions alongside the USDA SFSP. Delivered by camp NERF counsellors with peer mentors and parent engagement strategies. Dose: Day program (6 h/day, 5 days/w), 8 w duration (64 h camp exposure per child)	Mental health: Positive and negative affect (Positive and Negative Affect Survey) *(1)*
Levy [59] (2020) USA	Three group within subject, repeat measure (no control)	High-school-aged adolescents (14–17 years) from Latinx, Black and multi-racial backgrounds. n = 18 (44%F) Mean age: not reported	Goal: Address stress, anxiety and depression Description: Hip-hop-focused program, based around mix-tape creation using three different leadership styles. Facilitated by a school counselor and social worker with hip-hop experience Dose: Delivered within an existing six w summer day camp setting. Program delivered for approx. 2 h/day, 5 days for 1 w	Mental health: Perceived stress (The Perceived Stress Scale), depression, and anxiety (Abbreviated Brief Symptom Inventory) *(3)*
Manjunath [57] (2004) India	Non-randomized controlled study	11–16-year-old school children n = 90 (48%F) Mean age: not reported	Goal: Improve memory through yoga training and creative activities Description: Fine arts (e.g., drama, cricket, dance, singing, pottery) and yoga classes with relaxation and meditation. Dose: Format not reported, 8 h/day, 10-day duration	Cognitive: Memory: Spatial (geometric shapes) and verbal (nonsense syllables) (outcomes devised by study authors) *(2)*
Nabors [68] (2001) USA	Within subject, repeat measure (no control)	Children aged 5–11 from homeless or very low-income families. n = 53 (45%F) Mean age: 9.4	Goal: To support mental health and enhance coping strategies and social skills of children and enhance parenting skills (discipline and problem behaviors) Description: Health prevention (hygiene, diet, exercise, rest), mental health support (e.g., coping and drug use), individual therapy (psychological or adjustment issues), enrichment, parent training sessions. Provided by teachers, mental health clinicians. Dose: Day program, 5–6 w duration.	Social: Child’s behavioral, emotional and academic functioning relative to peers (Parents: How My Child Is Doing Survey, Teachers: Teacher Survey of Student Progress) *(2)*
Pierce [69] (2017) USA	Within subject, repeat measure (no control)	Grade-9–10 African American students from low-income communities. n = 36 (%F not reported) Mean age: 14.9 (yr1), 15.5 (yr2)	Goal: Provide youth with an integrative health education in the context of environment and community Description: Partnership between local government and private organization. Mission Thrive Summer program ran over two consecutive summers. Activities included: nutrition, cooking, farming, physical activity, and leadership delivered by program leaders, exercise instructors, and older peers. Dose: Day program (7.5 h/day), 5 days/w, 6 w duration (total 30 sessions)	Mental health: Stress (Perceived Stress Scale) Other: Mindfulness (Child and Adolescent Mindfulness Measure) *(3)*
Pradhan [60] (2009) India	Within subject, repeat measure (no control)	English-speaking adolescents aged 13–16 years n = 133 (38%F) Mean age: 14 (1)	Goal: Personality development program Description: Yoga training including meditation, relaxation, chanting, and yoga postures Dose: Format not reported. 10 consecutive days	Cognitive: Memory and selective attention (Digit–Letter Substitution Task) *(3)*
Readdick [70] (2005) USA	Within subject, repeat measure (no control)	6–12-year-old, low-income, at-risk children n = 78 (49%F) Mean age: not reported	Goal: Increase self-esteem of low-SES, school-aged children Description: Activities within nature settings inc. hiking, camping outside, interactions with animals, playground games. Led by camp counsellors and graduate students. Dose: 12-day residential program	Self-perception: Self-Concept (Piers-Harris Children’s Self-Concept Scale: total score, popularity, physical, intellectual, happiness, behavior, and anxiety) *(3)*
Riley [71] (2016) USA	Within subject, repeat measure (no control)	9–15-year-old vulnerable youth of color living in poverty. n = 329 (36%F) Mean age: 11.5 (1.5)	Goal: Promote social skills through sport amongst vulnerable youth Description: Social skills integrated into sport (e.g., basketball, lacrosse, and social dance) and play-based activities. Led by coaches. Dose: Day program (6 h/day), 19 days duration	Social: Social Skills (Social Skill Improvement System subscales: perceived self-control and perceived externalizing behaviors) *(3)*
Saunders-Ferguson [72] (2008) USA	Within subject, repeat measure (no control)	12–18-year-old adolescents registered in 4-H Horsemanship school n = 122 (89%F) Mean age: 14	Goal: Improve self-esteem, physical self-competence, and physical self-acceptance Description: Florida 4-H Horsemanship program delivered by experienced riders Dose: Residential program (5–7 h/day), 6 days, 1 w duration	Self-perception: Self-esteem (Rosenberg’s Self-Esteem Scale), physical competence, physical self-acceptance Other: Personal “Horsemanship Attributes” (responsibility, confidence, motivation) (Survey of Youth Participating in Equine Activities) *(3)*
Smith [58] (2022) USA	Within subject, repeat measure (3 arm, no control)	6^th^–8^th^-grade children from racial/ethnic minority backgrounds or low-SES families n = 51(53%F) Mean age: not reported	Goal: support social–emotional learning, mental health and well-being Description: Comparison of three various programs: Experimental Education, Experiential Camp, Recreational camp. Activities included day and overnight hikes, adventure activities, canoeing, lessons about vision and action, grit and resilience and integrity, mental health, respect and communication. Programs delivered at different sites/times. Dose: Residential program, 4–8 days	Mental health: Life Satisfaction (Brief Multidimensional Student’s Life Satisfaction Scale), Positive and Negative Affect (Positive and Negative Affect Survey) Social: Social and leadership Skills (perseverance, teamwork, and social skills) (developed by study authors) Cognitive: Executive Functioning: Plan Management (revised Executive Skills Questionnaire) *(3)*
Thurber [73] (2006) USA	Within subject, repeat measure (no control)	8–14-year-old children attending nationally accredited summer camp across various sites. n = 3395 (64%F) Mean age: 11.1 (1.9)	Goal: Determine how camp experiences influence child development outcomes Description: ACA accredited programs (various sites), mixed curriculums and delivery methods Dose: Day and residential programs, 1–4 + w duration (minimum of 1 session/w)	Self-perception: Self-esteem, positive identity (independence) Social: Social skills (leadership, friendship, social comfort, and peer relationships) Other: Character skills (adventure and exploration; and environmental awareness), values and Spirituality (Caper Growth Index Child Form Sub-Domains) *(3)*
Travis [74] (2019) USA	Within subject, repeat measure (no control)	11–15-year-old children from low-SES and racially/ethnically diverse backgrounds. n = 35 (57%F) Mean age: 12.5	Goal: To improve positive youth development and decrease depression and anxiety symptoms Description: Educational and mental health-focus program included Hip Hop, Empowerment, and Therapeutic Beat Making strategies. Led by teachers and staff from partner organizations Dose: Day program (full day), 5 days/w, 6 w duration	Mental health: Depression and anxiety (Abbreviated Brief Symptom Inventory) Self-perception: Confidence (Self-Efficacy Scale), competence (subset: Self-Liking/Self Competence Scale) Social: Connection, caring (Vaux Social Support Record, Measure of Emotional Empathy for Adolescents and Adults), citizenship (subset: theoretical model of Active and Engaged Citizenship), community (Sense of Community Index 2) Other: Character/delinquency (subscale: Pittsburgh Youth Study Attitude Towards Delinquency) *(3)*
Ullrich-French [75] (2012) USA	Within subject, repeat measure (no control)	9–16-year-old youth with overweight/obesity from diverse ethnic and low-SES families.n = 197 (52%F) Mean age: 11.8 (1.6)	Goal: Increase in psychological outcomes and changes in perceptions of social connection Description: Sports, physical activity, and classroom activities (e.g., computers/writing and health education). Led by university students (student athletes and PE students). Dose: Day program (6 h/day), 5 days/w, 4 w duration	Self-perception: Global self-worth, social, scholastic, and physical/athletic competence (Self-Perception Profile for Children), hope (Children’s Hope Scale) *(3)*
Werch [53] (2000) USA	Cluster RCT (three arms)	Grade-7–9 students in urban, suburban, and rural settings. n = 178 (48%F) Mean age: 13.1 (1)	Goal: Risk education for alcohol use/abuse Description: STARS for Families program to provide ongoing alcohol prevention advice and education for parents. Intervention modified for phone delivery using a standardized protocol. Dose: Initial visitx1 (school), phone consults with trained RN (home based). Information cards mailed 2 x/w. 5 w duration	Other: Substance use: % of children using alcohol (The Youth Alcohol and Drug Survey) *(1)*
Wong [76] (2009) USA	Within subject, repeat measure (no control)	10–14-year-old children with obesity from multiethnic and diverse socioeconomic backgrounds. n = 21 (76%F) Mean age: 11.4 (1.4)	Goal: Improve self-esteem and promote a long-term healthy lifestyle Description: Program: Kamp K’aana. Behavioral lessons (e.g., journaling and goal setting), physical activity (e.g., aerobic exercise), nutrition classes with parent engagement component. Led by camp counsellors. Dose: Residential program: 2 w duration	Self-perception: Self-worth, competence (scholastic, social, athletic competence), physical appearance Social: Behavioral conduct (Self-Perception Profile for Children) *(3)*

Intervention description: Goal: the skills, outcomes, or behaviors targeted by the program. Description: curriculum and delivery features of the program. Dose: format (day/residential), number of program hours per day, days per week, and total week duration. Key: ACA, American Camp Association; CATCH, Coordinated Approaches to Child Health; COPE, Creating Opportunities for Personal Empowerment; CHAMPS, Children and Mentally Ill Parents; h, hours; K, kindergarten; NERF, Nutrition Education Recreation and Fitness (camp); OCEBM, Oxford Centre for Evidence Based Medicine Levels of Evidence (2011 (Levels A–D)) [50]; PA, physical activity; PE, physical education; RCT, randomized controlled trial; RN, registered nurse; SES, socioeconomic status; SFSP, Summer Food Service Program; STARS, Start Taking Alcohol Risks Seriously; USA, United States; USDA, United States Department of Agriculture.

**Table 2:** Summary of participant and program characteristics.

Study	Participant Characteristics		Program Characteristics								
First Author, (Year)	Age	SES	Format	Setting	Content	Cost	Duration (W)	Daily Contact	Intensity	Enrichment	Attendance
Anderson-Butcher 2013 [61]	Middle school	NR	Day	School	Sport-based youth development program	NR	4	Half day	Daily	Sports, health/wellness, drug/alcohol prevention.	NR. Transport provided.
Ay 2019 [62]	Primary school	NR	Day	School	Conflict resolution	Funded	6	Sessional within a school day program	Daily		NR
Bethea 2012 [63]	Primary school	Low	Day	School	General	NR	6	Full day	Daily	Games, activities, food, arts, sports, field trips	High
delosPinos 2020 [64]	High school	Low	NR	NR	Social skills	Funded	2	NR	Daily	Games, gymkhanas, workshops, films, walks	NR
Exner-Cortens 2020 [54]	High school	Mixed	Day	School	Risk behavior and mental health	Funded	2	Half day	Daily		High
Fainardi 2020 [65]	Mixed	NR	Day	NR	General	NR	2	Full day	Daily	Sports, recreation, music, arts, cooking, nature parks	High
Fujieda 2011 [77]	Primary school	NR	Home	Home	Communication skills	NR	NR	Sessional	Occasional		Low
Gately 2005 [55]	Mixed	Mixed	Residential	School	Physical activity and weight control	Paid	2 to 6	Full day	Daily	Fun land- and water-based physical activities	NR
Gerber 2022 [56]	Mixed	NR	Day	Mixed	NR	NR	NR	NR	Daily		NR
Goodyear 2009 [66]	Primary school	NR	Day	NR	Risk behavior, mental health	NR	1	Half day	Daily		NR
Henert 2021 [67]	Mixed	NR	Day	School	General	Paid or part-paid (scholarship)	6	School day	Daily	Sport	Moderate
Hopkins 2019 [52]	Primary school	Low	Day	School	Physical and mental health	Funded	8	School day	Daily		Varied by site (low to high)
Levy 2020 [59]	High school	Racial minority	Day	School	General with stress reduction strategies	NR	1 to 6	Sessional within a full day program	Daily	Music	NR
Manjunath 2004 [57]	Mixed	NR	NR	Private	Yoga and fine arts	NR	1.5	NR	Daily	Arts, drama, games, sport, yoga, relaxation, meditation	NR
Nabors 2001 [68]	Primary school	Low	Day	School and community partnership	Mental health	Funded	5 to 6	School day	Daily	Art, dance, recreational activities, field trips	NR
Pierce 2017 [69]	High school	Low	Day	Private and community partnership	African American focused enrichment	Funded	6	Full day	Daily	Cooking, farming, sport, yoga, breathing, relaxation, nature play	High
Pradhan 2009 [60]	High school	NR	NR	Private	Yoga	NR	1.5	Full day	Daily	Yoga, meditation, relaxation	NR
Readick 2005 [70]	Primary school	Low	Residential	Private	General	Partially funded	2	Full day	Daily	Nature plan, hiking, camping, animals, playground	High
Riley 2016 [71]	Middle school	Low	Day	NR	Sport based youth program	Funded	3	School day	Daily	Sports.	High. Transport provided
Saunders-Ferguson 2008 [72]	High school	NR	Residential	Private	Enrichment: horseman-ship activities	Paid	1	Full day	Daily	Horse-riding	NR
Smith 2022 [58]	Middle school	Low	Residential	Community	General	Funded	1	Full day	Daily	Recreation, hiking, camping. water and land-based sports and activities.	NR
Thurber 2006 [73]	Middle school	Mixed	Mixed	Mixed	Mixed	Funded	1 to 4	NR	Daily		High
Travis 2019 [74]	Middle school	Low	Day	School	Mental health subgroup within general program	NR	6	Sessional within a full day general program	Daily	Music	NR
Ullrich-French 2013 [75]	Middle school	Low	Day	School	Physical activity	Funded	5	Half day	Daily	Sport, physical activity, recreation, computers, writing	NR
Werch 2020 [53]	Middle school	NR	Home	Home	Risk behavior, mental health	Funded	NR	Sessional	Occasional		NR
Wong 2009 [76]	Middle school	Mixed	Residential	Private	Physical and mental health	Mixed (Paid for some, Others on scholarships)	2	Full day	Daily		NR

Legend: NR: not reported. W: weeks. Participant characteristics. Age: range and mean values for the majority of participants. Primary school, kindergarten/reception to grade six (ages five to 11 years); middle school, grades 7–9 (ages 12–14 years); high-school students, grades 10–12 (ages 15 years and older). SES: socioeconomic status. Program characteristics. Format: residential (overnight stays), day (daytime only programs run outside the home), mixed (more than one setting). Setting: community (e.g., public spaces, non-profit community organizations), school (educational settings inc. schools and universities). Content: general (enrichment and recreation-based programs). Cost: free (no cost for participants), funded (payment made on behalf of the participants, e.g., trial funding), paid (participants paid attendance fees). Daily contact, contact hours per session: full day (7+ hours and residential programs), school day (6–7 h), half day (2–6 h), sessional (2 h or less). Intensity, frequency of sessions: occasional, less than once per week (e.g., home-based program with formal contact sessions at the start and end of the program). Attendance (% of scheduled sessions attended): up to 50% (low), 51–65% (moderate), 66%+ high. Primary-school children, kindergarten/reception to grade six (ages five to 11 years); middle-school students, grades 7–9 (ages 12–14 years); high-school students, grades 10–12 (ages 15 years and older).

## Data Availability

The datasets analyzed in this review are derived from publicly available sources. All data can be accessed through the original publications.

## Supplementary Materials

The following supporting information can be downloaded at https://www.mdpi.com/article/10.3390/children11080887/s1. File S1: Inclusion-exclusion criteria; File S2: Search strategy; File S3: Example data extraction form; File S4: Reasons for exclusion table; File S5: Thematic Coding of Study and Program Characteristics; File S6: Mental Health Meta analysis with sensitivity; File S7: Mental health subgroup analyses.
