# Supplementary material for: Effect of Summer Holiday Programs on Children’s Mental Health and Well-Being: Systematic Review and Meta-Analysis
Source: Children (Basel). 2024 Jul 23;11(8):887. doi: 10.3390/children11080887 (PMC11352663; doi:10.3390/children11080887)
Supplement: Supplementary file 1 [file children-11-00887-s001.zip › File S1. Inclusion-exclusion criteria.pdf]

Supplementary File S1. Inclusion/exclusion criteria.

Overview of the inclusion/exclusion criteria using the PICO framework

	**Inclusion**	**Exclusion**
Populations/ participants	Children in years kindergarten to grade 12	Medical populations (e.g. diabetes, cancer), special needs (learning or intellectual disabilities), gifted/specially talented.
Intervention	Summer holiday programs: Programs of at least five days duration conducted over the summer holiday period. Programs may include day or overnight or residential programs.	Program evaluation with no mental health outcomes.
Comparators	Control group not receiving a summer program.	
Outcome	1. Mental health (e.g. psychological wellbeing, depression)<br>2. Cognitive (e.g. working memory, executive function)<br>3. Social and emotional (e.g. social connectedness, sadness, loneliness, appropriate behaviour)	1. Academic performance
Study designs	1. Randomized and non-randomized, controlled trials.<br>2. Quasi-experimental: single group pre- post-intervention designs	
Publication types	Full length, peer-reviewed original research articles published in/after the year 2000. No language limits.	Grey literature, conference abstracts, dissertations. Studies published prior to 2000. Review papers
