# Supplementary material for: Effect of Summer Holiday Programs on Children’s Mental Health and Well-Being: Systematic Review and Meta-Analysis
Source: Children (Basel). 2024 Jul 23;11(8):887. doi: 10.3390/children11080887 (PMC11352663; doi:10.3390/children11080887)
Supplement: Supplementary file 1 [file children-11-00887-s001.zip › File S2. Search strategy.pdf]

Supplementary File S2. Search strategy.

Medline (OVID)	1	Child/
	2	Adolescent/
	3	(child* or adolescen* or teen* or youth* or young people or young person or boy or girl).ti,ab,kf.
	4	Holidays/
	5	((summer or school) adj2 (holiday* or vacation*)).ti,ab,kf.
	6	1 OR 2 OR 3
	7	4 OR 5
	8	6 AND 7
	9	limit 9 to yr="2000 -Current"
PsychINFO (OVID)	1	Adolescent Behavior/ or Adolescent Attitudes/ or Adolescent Psychology/ or Adolescent Health/
	2	Child Care/ or Child Behavior/ or Child Attitudes/ or Child Psychology/ or Child Health/
	3	child.mp.
	4	adolescent.mp.
	5	(child* OR adolescen* OR boy OR girl).ti,ab,tw.
	6	1 OR 2 OR 3 OR 4 OR 5
	7	Holidays/
	8	((school* or summer*) adj2 (holiday* or vacation*)).ti,ab,tw.
	9	(summer adj2 (months or period or learning or "between grade*")).ti,ab,tw.
	10	7 OR 8 OR 9
	11	6 AND 10
	12	limit 11 to yr="2000 -Current"
Embase	1	school child/
	2	adolescent/
	3	(child* or adolescen*).ti,ab,kf.
	4	1 or 2 or 3
	5	((Summer* OR school*) adj2 (holiday* OR Vacation*)).ti,ab,kf.
	6	(summer adj2 (months OR period OR learning OR "between grade*")).ti,ab,kf.
	7	5 OR 6
	8	4 AND 7
	9	limit 8 to yr="2000 -Current"
JBI	1	(child* or adolescen* or student* or grade* or boy* or girl* or youth).mp.
	2	(Holiday* or vacation* or summer*).mp.
	3	1 and 2
	4	limit 3 to yr="2000 -Current"

ERIC	(((mainsubject(Child) OR mainsubject(Adolescent) OR mainsubject(Youth)) OR (TI,AB,IF(Children OR child* OR adolescen* OR "boy" OR "girl") OR TI,AB,IF(student* OR Grade*) OR TI,AB,IF(youth))) AND (mainsubject(vacations) OR TI,AB,IF((school*) NEAR/2 (Vacation* OR holiday* OR Summer*)) OR TI,AB,IF((summer) NEAR/2 (Vacation* OR months OR holiday OR period OR school)))) AND (pd(20000101-20230331) AND PEER(yes))
Scopus	(TITLE-ABS-KEY (child* OR adolescen* OR boys OR girls)) AND (TITLE-ABS-KEY ((summer* W/2 (holiday* OR vacation* OR school*)))) AND PUBYEAR > 1999 AND PUBYEAR < 2024 AND (LIMIT-TO (DOCTYPE , "ar"))

Search strategy:
https://www.cabidigitallibrary.org/doi/10.1079/searchRxiv.2023.00381
