# Supplementary material for: Effect of Summer Holiday Programs on Children’s Mental Health and Well-Being: Systematic Review and Meta-Analysis
Source: Children (Basel). 2024 Jul 23;11(8):887. doi: 10.3390/children11080887 (PMC11352663; doi:10.3390/children11080887)
Supplement: Supplementary file 1 [file children-11-00887-s001.zip › File S3. Example data extraction form.pdf]

Supplementary File S3. Example data extraction form.

Study title	
Year of publication	
Lead author email address	
Country in which the study was conducted	
Reviewer	
Study design	

Population description

	sample size n=	sex female n=	female % (whole number)	range (years)	age mean (years)	Age (SD) (years)
Whole sample						
Intervention group						
Alternative intervention group (if applicable)						
Control group						

Program Features

Method of recruitment of participants	
Population SES	
Goal of the program	
Intervention Program Description	
What did the comparison group receive?	

Program structure – setting and delivery

Intervention group

Setting	
Delivery	
Cost	
Format: day/residential	
Duration (days/week, total weeks from start to finish)	
Duration per day (hrs)	
Number of sessions	
Other program features	

Outcome measured & tool used

- ☐ Physical Health: ___________________________
- ☐ Health Behaviour: _________________________

Physical Health Outcome

	Outcome	N=	baseline mean (variability metric and value)	end program mean (variability metric and value)	Effect size, test statistic and value	confidence intervals (lower and upper)	Follow-up measures? (timepoint)	Follow-up outcomes (change, effect, significance, CI)
Intervention group								
Control group								

Notes

Health Behaviour Outcome

	Outcome	N=	baseline mean (variability metric and value)	end program mean (variability metric and value)	Effect size, test statistic and value	confidence intervals (lower and upper)	Follow-up measures? (timepoint)	Follow-up outcomes (change, effect, significance, CI)
Intervention group								
Control group								

Notes

Subgroup analyses by SES or race (& results)?

Implementation outcomes

	Attendance level	Incentives to attend	Adverse events	Funding for the program	Conflicts of interests
Intervention group					
Alternative intervention (if applicable)					

Any notes you would like the other authors to know?

Summarize the paper's main findings:
