# Supplementary material for: Effect of Summer Holiday Programs on Children’s Mental Health and Well-Being: Systematic Review and Meta-Analysis
Source: Children (Basel). 2024 Jul 23;11(8):887. doi: 10.3390/children11080887 (PMC11352663; doi:10.3390/children11080887)
Supplement: Supplementary file 1 [file children-11-00887-s001.zip › File S4. Reasons for exclusion table.pdf]

Supplementary File S4. Reasons for study exclusion.

Title	Authors	Year Published	Exclusion Reason
A comparison of a gardening and nutrition program with a standard nutrition program in an out-of-school setting	Poston, S. A.', 'Shoemaker, C. A.', 'Dzewaltowski, D. A.'	2005	Wrong intervention
A Creative Strengths-Based Group Counseling Intervention for African American Boys	Prasath, P. R.', 'Steen, S.', 'McVay, K.'	2023	Wrong outcomes
A fact-finding study concerning the pro-environmental behavior of elementary school students and guardians: First analysis of participants in Kyoto Prefecture Energy Conservation Challenge during summer vacation	Kim, Y.', 'Kihara, H.', 'Matsubara, N.'	2019	Wrong outcomes
A multi-method analysis of body mass index, physical activity, and executive functions among urban minority girls	Ward, Amanda K.'	2016	Wrong outcomes
A partnership approach to tackling inequalities through a summer holiday enrichment programme	Holmes, E.', 'Palmer, K.'	2016	Wrong literature type
A pilot study evaluating the feasibility of a summer gardening program to prevent summer weight gain in overweight adolescents	Jacquart, S. R.', 'Schoeller, D. A.', 'Adams, A. K.', 'Larson, N.', 'Dennis, S. F.', 'LaRowe, T. L.', 'Carrel, A. L.'	2010	Wrong literature type
A Professional Development School--Sponsored Summer Program for At-Risk Secondary Students	Cuddapah, Jennifer L.', 'Masci, Frank J.', 'Smallwood, Jo Ellen', 'Holland, Jennifer'	2008	Wrong outcomes
A rationale for the use of anthropometric measurements and bioelectrical impedance analysis as efficacy criteria for summer camp healthcare	Gavryushin, M. Yu', 'Sazonova, O. V.', 'Gorbachev, D. O.', 'Borodina, L. M.', 'Frolova, O. V.', 'Tupikova, D. S.'	2019	Wrong study design
A residential summer camp can reduce body fat and improve health-related quality of life in obese children	Wong, William W. Barlow, Sarah E. Mikhail, Carmen Wilson, Theresa A. Hernandez, Paula M. Shypailo, Roman J. Abrams, Stephanie H.	2013	Wrong outcomes

A summer nutrition benefit pilot program and low-income children's food security	Collins, Ann M.', 'Klerman, Jacob A.', 'Briefel, Ronette', 'Rowe, Gretchen', 'Gordon, Anne R.', 'Logan, Christopher W.', 'Wolf, Anne', 'Bell, Stephen H.'	2018	Wrong outcomes
Active generations: An intergenerational approach to preventing childhood obesity	Werner, D.', 'Teufel, J.', 'Holtgrave, P. L.', 'Brown, S. L.'	2012	Wrong study design
Active summers matter: evaluation of a community-based summertime program targeting obesogenic behaviors of low-income, ethnic minority girls	Bohnert, A. M.', 'Ward, A. K.', 'Burdette, K. A.', 'Silton, R. L.', 'Dugas, L. R.'	2014	Wrong outcomes
Addressing Childhood Hunger during the Summer Months: Using Gleaned Produce for Snacks and Interactive Nutrition Education on Food Systems and Healthy Eating	Oo, K.', 'Stephenson, T.', 'Hege, A.', 'Brewer, D.', 'Gamboa, L.', 'Hildesheim, L.', 'Serra, L.', 'Houlihan, J.', 'Koempel, A.'	2020	Wrong outcomes
An evaluation of a parent tutoring reading fluency program	Mitchell, Rachel Courtney'	2013	Wrong literature type
An exploratory study comparing students' science identity perceptions derived from a hands-on research and nonresearch-based summer learning experience	Hernandez-Matias, L.', 'Perez-Donato, L.', 'Roman, P. L.', 'Laureano-Torres, F.', 'Calzada-Jorge, N.', 'Mendoza, S.', 'Washington, A. V.', 'Borrero, M.'	2020	Wrong outcomes
An integrated components preventive intervention for aggressive elementary school children: The early risers program	August, G. J.', 'Realmuto, G. M.', 'Hektner, J. M.', 'Bloomquist, M. L.'	2001	Wrong population
An urban garden initiative: A component of project healthy schools	Wei, W. C. R.', 'Heeres, A.', 'Aaronson, S.', 'Rogers, R.', 'Lee, A.', 'Pew, A.', 'Foti, A.', 'Vuong, B.', 'Corriveau, N.', 'Jiang, Q.', 'Kline-Rogers, E.', 'Goldberg, C.', 'DuRussel-Weston, J.', 'Jackson, E. A.', 'Eagle, K. A.'	2014	Wrong literature type
Camp Jump Start: Effects of a Residential Summer Weight-Loss Camp for Older Children and Adolescents	Huelsing, Jean Kanafani, Nadim Mao, Jingnan White, Neil H.	2010	Wrong outcomes

Camp NERF: Caregiver outcomes from a theory-based nutrition education recreation and fitness program aimed at preventing unhealthy weight gain in underserved children during summer months	Hopkins, L. C.', 'Webster, A.', 'Sharn, A.', 'Gunther, C.'	2017	Wrong literature type
Camp NERF: Efficacy of a Theory-Based Nutrition Education Recreation and Fitness Program Aimed at Preventing Unhealthy Weight Gain in Disadvantaged Children during Summer Months	Hopkins, Laura Gunther, Carolyn	2016	Wrong literature type
Camp NERF: Feasibility, Acceptability, and Potential Efficacy of a Theory-Based Nutrition Education Recreation and Fitness Program Aimed at Preventing Unhealthy Weight Gain in Disadvantaged Children during Summer Months	Hopkins, Laura Rose, Angela Gunther, Carolyn	2015	Wrong literature type
Camp NERF: methods of a theory-based nutrition education recreation and fitness program aimed at preventing unhealthy weight gain in underserved elementary children during summer months	Hopkins, L. C.', 'Fristad, M.', 'Goodway, J. D.', 'Eneli, I.', 'Holloman, C.', 'Kennel, J. A.', 'Melnyk, B.', 'Gunther, C.'	2016	Wrong literature type
Camp-Based Immersion Treatment for Obese, Low Socioeconomic Status, Multi-Ethnic Adolescents	Carraway, Marissa Lutes, Lesley D. Crawford, Yancey Pratt, Keeley J. McMillan, Amy Gross Scripture, Lee G. Henes, Sarah T. Cox, James Vos, Paul Collier, David N.	2014	Wrong outcomes
Can an Immersion in Wellness Camp Influence Youth Health Behaviors?	Mabary-Olsen, Elizabeth A.', 'Litchfield, Ruth E.', 'Foster, Randal', 'Lanningham-Foster, Lorraine', 'Campbell, Christina'	2015	Wrong outcomes
Changes in Daily Step Counts and Health-Related Fitness after a Sports-Based Residential Summer Camp in Boys	Wahl-Alexander, Zachary', 'Brusseau, Timothy', 'Burns, Ryan'	2020	Wrong outcomes
Characteristics of effective summer learning programs in practice	Bell, S. R.', 'Carrillo, N.'	2007	Wrong literature type

Children's Moderate to Vigorous Physical Activity Attending Summer Day Camps	Brazendale, Keith Beets, Michael W. Weaver, R. Glenn Chandler, Jessica Randel, Allison B. Turner-McGrievy, Gabrielle Moore, Justin B. Huberty, Jennifer Ward, Dianne S.	2017	Wrong study design
Cognitive outcomes from the Game-Design and Learning (GDL) after-school program	Akcaoglu, M.', 'Koehler, M. J.'	2014	Wrong intervention
Combating Child Summer Food Insecurity: Examination of a Community-Based Mobile Meal Program	Bruce, J. S.', 'De La Cruz, M. M.', 'Lundberg, K.', 'Vesom, N.', 'Aguayo, J.', 'Merrell, S. B.'	2019	Wrong outcomes
Come together, play, be active: Physical activity engagement of school-age children at Play Streets in four diverse rural communities in the U.S	Umstattd Meyer, M. R.', 'Bridges Hamilton, C. N.', 'Prochnow, T.', 'McClendon, M. E.', 'Arnold, K. T.', 'Wilkins, E.', 'Benavidez, G.', 'Williams, T. D.', 'Abildso, C. G.', 'Pollack Porter, K. M.'	2019	Wrong outcomes
Comparing Campers' Physical Activity Levels Between Sport Education And Traditional Instruction in a Residential Summer Camp	Wahl-Alexander, Zachary Morehead, Craig A.	2017	Wrong outcomes
Comparison of development of physical fitness parameters in different summer school programs	Güngör, E.', 'Onurcerrah, A.', 'Çobano?lu, H.', 'Kaço?lu, C.', 'Yilmaz, I.'	2010	Wrong outcomes
Complete EATS: Summer meals offered by the emergency department for food insecurity	Cullen, D.', 'Blauch, A.', 'Mirth, M.', 'Fein, J.'	2019	Wrong setting
Computing goals, values, and expectations: Results from an after-school program for girls	Denner, J.', 'Werner, L.', 'Martinez, J.', 'Bean, S.'	2012	Wrong outcomes
Contribution of children's reading motivation and prosocial efficacy to reading growth	Ha, C.', 'Roehrig, A. D.'	2022	Wrong outcomes
Culturally relevant reading for supporting underserved children's prosocial self-efficacy and reading skills: A transformative social-emotional learning approach	Ha, Cheyeon'	2023	Wrong literature type

Development and evaluation of WillTry. An instrument for measuring children's willingness to try fruits and vegetables	Thomson, J. L.', 'McCabe-Sellers, B. J.', 'Strickland, E.', 'Lovera, D.', 'Nuss, H. J.', 'Yadrick, K.', 'Duke, S.', 'Bogle, M. L.'	2010	Wrong outcomes
Diet of children under the government-funded meal support program in Korea	Kwon, Sooyoun', 'Lee, Kiwon', 'Yoon, Jihyun'	2010	Wrong intervention
Digital Game Building: Learning in a Participatory Culture	Li, Qing'	2010	Wrong study design
Do you hear what I hear? Overweight children's perceptions of different physical activity settings	Meaney, K. S.', 'Hart, A. M.', 'Griffin, L. K.'	2011	Wrong outcomes
Effect of sleep intervention using a summer holiday workbook for junior high-school students	Oka, Y.', 'Horiuchi, F.', 'Kawabe, K.'	2015	Wrong literature type
Effectiveness of an intervention program for six year olds: A summer-school model	Bekman, S.', 'Aksu-KoÃ§, A.', 'Erguvanli-Taylan, E.'	2012	Wrong outcomes
Effectiveness of an intervention program for six-year-olds: A summer-school model	Bekman, S.', 'Aksu-KoÃ§, A.', 'Erguvanli-Taylan, E.'	2011	Wrong intervention
Effectiveness of the bicisalud programme in a group of schoolchildren with excess weight	Perez, E. R. S.', 'Aranzamendi, J. I. L.', 'Cano, M. R. M.', 'Delgado, D. B.', 'CÃ¡mara, G. L. C.'	2018	Wrong outcomes
Effects of a practice-focused nutrition intervention in Hungarian adolescents	Takacs, H.', 'Martos, E.', 'Anna Kovacs, V.'	2020	Wrong intervention
Effects of a summer school-readiness programme on measures of literacy and behaviour growth: A pilot study	McLeod, Ragan', 'Kim, Sunyoung', 'Tomek, Sara', 'McDaniel, Sara'	2019	Wrong population
Effects of pairing aggressive and nonaggressive children in strategic peer affiliation	Hektner, J. M.', 'August, G. J.', 'Realmuto, G. M.'	2003	Wrong outcomes
Effects of Recreational exercises on Children's Anthorpometric Parameters and their level of nutrition knowledge	Karacabey, K.', 'Derdin, M.'	2014	Wrong outcomes
Effects of summer school participation and psychosocial outcomes on changes in body composition and physical fitness during summer break	Park, Kyung-Shin; Lee, Man-Gyoon	2015	Wrong outcomes
Engaging Excellent Aboriginal Students in Science: An Innovation in Culturally-Inclusive Schooling	Aldous, Carol', 'Barnes, Alan', 'Clark, Julie'	2008	Wrong outcomes

Environmental and social-motivational contextual factors related to youth physical activity: Systematic observations of summer day camps	Zarrett, N.', 'Sorensen, C.', 'Skiles, B.'	2013	Wrong study design
Evaluation of a community-based intervention to promote physical activity in youth: Lessons from active winners	Pate, R. R.', 'Saunders, R. P.', 'Ward, D. S.', 'Felton, G.', 'Trost, S. G.', 'Dowda, M.'	2003	Wrong outcomes
Evaluation of a five-day recipe booklet for enhancing the use of fruits and vegetables in low-income households	Birmingham, Brenda', 'Shultz, Jill Armstrong', 'Edlefsen, Miriam'	2004	Wrong outcomes
Examining the impact of a summer learning program on children's weight status and cardiorespiratory fitness: A natural experiment	Hunt, E. T.', 'Whitfield, M. L.', 'Brazendale, K.', 'Beets, M. W.', 'Weaver, R. G.'	2019	Wrong study design
Examining the potential protective effect of structured programming on child weight during the summer months through intervention and observational research: Camp NERF (nutrition, education, recreation, and fitness) and project SWEAT (summer weight and environmental assessment trial)	Hopkins, Laura C.'	2019	Wrong literature type
Experiencing nature in children's summer camps: Affective, cognitive and behavioural consequences	Collado, Silvia', 'Staats, Henk', 'Corraliza, Jose A.'	2013	Wrong outcomes
Familias Unidas: The efficacy of an intervention to promote parental investment in Hispanic immigrant families	Pantin, Hilda', 'Coatsworth, J. Douglas', 'Feaster, Daniel J.', 'Newman, Frederick L.', 'Briones, Ervin', 'Prado, Guillermo', 'Schwartz, Seth J.', 'Szapocznik, Jose'	2003	Wrong intervention
Families, Schools, and Summer Learning	Borman, Geoffrey D.', 'Benson, James', 'Overman, Laura T.'	2005	Wrong outcomes
First year physical activity findings from turn up the HEAT (Healthy Eating and Activity Time) in summer day camps	Weaver, R. Glenn Brazendale, Keith Chandler, Jessica Turner-McGrievy, Gabrielle Moore, Justin B. Huberty, Jennifer Ward, Dianne S. Beets, Michael W.	2017	Wrong outcomes
Food Insecurity: A Constant Factor in the Lives of Low-Income Families in Scotland and England	Shinwell, Jackie', 'Defeyter, Margaret Anne'	2021	Wrong population

Food safety and sustainable nutrition workshops: Educational experiences for primary school children in Turin, Italy	Traversa, A.', 'Adriano, D.', 'Bellio, A.', 'Bianchi, D. M.', 'Gallina, S.', 'Ippolito, C.', 'Romano, A.', 'Durelli, P.', 'Pezzana, A.', 'Decastelli, L.'	2017	Wrong intervention
For comparison: experience with a children's obesity camp	Cooper, Christopher Sarvey, Sharon I. Collier, David N. Willson, Charles F. Green, Ira Pories, Mary Lisa Rose, Mary Ann Escott-Stump, Sylvia Pories, Walter J.	2006	Wrong study design
Fostering Healthy Development among Middle School Females: A Summer Program	Caton, Mary', 'Field, Julaine E.', 'Kolbert, Jered B.'	2010	Wrong literature type
From frontal teaching to emotional understanding - A modern concept in childhood obesity therapy	Katrin, H.', 'Dirk, B.', 'Anneco, D.', 'Bjorn, B.', 'Hanna, S.', 'Dagmar, L.'	2017	Wrong literature type
From Global to Local	Kye, Hannah'	2019	Wrong literature type
From I to We: Collaboration in Entrepreneurship Education and Learning?	Warhuus, Jan P.', 'Tanggaard, Lene', 'Robinson, Sarah', 'ErnÃ¸, Steffen Moltrup'	2017	Wrong population
Halting the Summer Achievement Slide: A Randomized Field Trial of the KindergARTen Summer Camp	Borman, Geoffrey D.', 'Goetz, Michael E.', 'Dowling, N. Maritza'	2009	Wrong outcomes
Healthy Lifestyle Fitness Camp: A summer approach to prevent obesity in low-income youth	George, Gretchen Lynn', 'Schneider, Constance', 'Kaiser, Lucia'	2016	Wrong outcomes
Healthy lifestyle intervention for obese children and their families as a part of the preventive health care programme for children and adolescents	Homsak, M.', 'Truden-Dobrin, P.', 'Vogrin, B.', 'Kotnik, P.', 'Pibernik, T.'	2021	Wrong literature type
Healthy Summer Learners: An explanatory mixed methods study and process evaluation	von Klinggraeff, L.', 'Dugger, R.', 'Brazendale, K.', 'Hunt, E. T.', 'Moore, J. B.', 'Turner-McGrievy, G.', 'Vogler, K.', 'Beets, M. W.', 'Armstrong, B.', 'Weaver, R. G.'	2022	Wrong outcomes

High intensity interval training vs. high-volume running training during pre-season conditioning in high-level youth football: a cross-over trial	Faude, O.', 'Schnittker, R.', 'Schulte-Zurhausen, R.', 'Muller, F.', 'Meyer, T.'	2013	Wrong population
High-intensity interval training for overweight adolescents: Program acceptance of a media supported intervention and changes in body composition	Herget, S.', 'Reichardt, S.', 'Grimm, A.', 'Petroff, D.', 'KÃ¤pplinger, J.', 'Haase, M.', 'Markert, J.', 'BlÃ¼her, S.'	2016	Wrong outcomes
Holiday Club Programmes in Northern Ireland: The Voices of Children and Young People	Shinwell, Jackie', 'Finlay, Ellen', 'Allen, Caitlin', 'Defeyter, Margaret Anne'	2021	Wrong study design
How physically active are children attending summer day camps	Beets, Michael W. Weaver, Robert G. Beighle, Aaron Webster, Collin A. Pate, Russell R.	2012	Wrong study design
Ignite the Leader Within: Virtual Latinx Youth Empowerment and Community Leadership amid COVID-19	Montes, Pablo', 'Bourommavong, Monica', 'Landeros, Judith', 'Urrieta, Luis, Jr.', 'Robinson, Courtney'	2021	Wrong outcomes
Impact of a year-round school calendar on children's BMI and fitness: Final outcomes from a natural experiment	Weaver, R. G.', 'Hunt, E.', 'Armstrong, B.', 'Beets, M. W.', 'Brazendale, K.', 'Turner-McGrievy, G.', 'Pate, R. R.', 'Maydeu-Olivares, A.', 'Saelens, B.', 'Youngstedt, S. D.', 'Dugger, R.', 'Parker, H.', 'von Klinggraeff, L.', 'Jones, A.', 'Burkhart, S.', 'Ressor-Oyer, L.'	2021	Wrong intervention
Impact of Citizenship Education on the Civic Consciousness of Nigerian Youth	Iyamu, Ede O. S.', 'Obiunu, Jude J.'	2005	Wrong outcomes
Impact of Year-Round and Traditional School Schedules on Summer Weight Gain and Fitness Loss	Brusseau, T. A.', 'Burns, R. D.', 'Fu, Y.', 'Glenn Weaver, R.'	2019	Wrong intervention
Implications of race and ethnicity for child physical activity and social connections at summer care programs	Prochnow, T.', 'Patterson, M. S.', 'Hartnell, L.', 'West, G.', 'Umstattd Meyer, M. R.'	2021	Wrong study design

Improving social skills in latency-age children with emotional disturbances through increased ethnic identity	Huey, Shontinese Cooper'	2006	Wrong literature type
Improving Urban Minority Girls' Health Via Community Summer Programming	Bohnert, Amy M. Bates, Carolyn R. Heard, Amy Burdette, Kimberly A. Ward, Amanda K. Silton, Rebecca L. Dugas, Lara R.	2017	Wrong study design
Increasing girls' physical activity during an organised youth sport basketball program: a randomised controlled trial protocol	Guagliano, Justin M.', 'Lonsdale, Chris', 'Kolt, Gregory S.', 'Rosenkranz, Richard R.'	2014	Wrong literature type
Increasing Physical Activity and Enjoyment Through Goal-Setting at Summer Camp	Wilson, Cait Sibthorp, Jim Brusseau, Timothy A.	2017	Wrong outcomes
Individual, social, physical environmental, and organizational correlates of children's summer camp-based physical activity	Hickerson, Benjamin D.'	2010	Wrong literature type
Influence of school holidays on weight of children participating in a tertiary hospital weight management programme	Rao, S.', 'Alexander, S.'	2011	Wrong literature type
Influence of Session Context on Physical Activity Levels Among Russian Girls During a Summer Camp	Guagliano, Justin M. Updyke, Natalie J. Rodicheva, Natalia V. Rosenkranz, Sara K. Dzewaltowski, David A. Schlechter, Chelsey R. Rosenkranz, Richard R.	2017	Wrong study design
Injury and Illness Benchmarking and Prevention for Children and Staff Attending U.S. Camps: Promising Practices and Policy Implications	Garst, Barry A.', 'Erceg, Linda E.', 'Walton, Edward'	2013	Wrong population
Investigation of the Effect of Regular Exercise on Some Motoric Features in Children in the 7-12 Age Group	Hazar, KÃ¼rsat'	2019	Wrong outcomes
Is a summer school programme a promising intervention in preparation for transition from primary to secondary school?	Siddiqui, N.', 'Gorard, S.', 'See, B. H.'	2014	Wrong outcomes
Learning problem-solving through making games at the game design and learning summer program	Akcaoglu, Mete'	2014	Wrong outcomes
Longitudinal achievement effects of multiyear summer school: Evidence from the Teach Baltimore randomized field trial	Borman, Geoffrey D.', 'Dowling, N. Maritza'	2006	Wrong outcomes

Making a Difference in Migrant Summer School: Testing a Healthy Weight Intervention	Kilanowski, J. F.', 'Gordon, N. H.'	2015	Wrong outcomes
Making the Most of School Vacation: A Field Experiment of Small Group Math Instruction	Schueler, Beth E.'	2020	Wrong outcomes
Maximizing children's physical activity using the LET US Play principles	Brazendale, Keith Chandler, Jessica Beets, Michael W. Weaver, Robert G. Beighle, Aaron Huberty, Jennifer Moore, Justin B.	2015	Wrong study design
Mental health awareness and mindfulness skills in primary school-aged children from ethnic minority backgrounds: A pilot health promotion programme	Aslam, A.', 'Hakim, A.', 'Ahmad, Z.'	2019	Wrong literature type
Metabolic health and academic achievement in youth at risk for high school dropout in rural Mississippi: The role of classroom management	Holmes, M. E.', 'Kvasnicka, M. A.', 'Brocato, D. K.', 'Webb, H. E.'	2018	Wrong intervention
Now We're All Family: Exploring Social and Emotional Development in a Summer Hip Hop Mixtape Camp	Travis, R., Jr.', 'Levy, I. P.', 'Morphew, A. C.'	2022	Wrong outcomes
Nutrition impacts in a randomized trial of summer food benefits to prevent childhood hunger in U.S. schoolchildren	Briefel, R. R.', 'Collins, A. M.', 'Wolf, A.', 'Gordon, A. R.', 'Cabili, C. L.', 'Klerman, J. A.'	2018	Wrong outcomes
Obesity and physical fitness of pre-adolescent children during the academic year and the summer period: Effects of organized physical activity	Christodoulos, Antonios D.', 'Flouris, Andreas D.', 'Tokmakidis, Savvas P.'	2006	Wrong study design
Opportunities for Promoting Youth Physical Activity: An Examination of Youth Summer Camps	Hickerson, Benjamin Henderson, Karla A.	2013	Wrong literature type
Opportunities for promoting youth physical activity: An examination of youth summer camps	Hickerson, Benjamin D.', 'Henderson, Karla A.'	2014	Wrong study design
Organizing Play Streets during school vacations can increase physical activity and decrease sedentary time in children	D'Haese, Sara, 'Van Dyck, Delfien', 'De Bourdeaudhuij, Ilse', 'Deforche, Benedicte', 'Cardon, Greet'	2015	Wrong outcomes
Outside-of-school time obesity prevention and treatment interventions in African American youth	Barr-Anderson, D. J.', 'Singleton, C.', 'Cotwright, C. J.', 'Floyd, M. F.', 'Affuso, O.'	2014	Wrong literature type

Parceling component effects of a multifaceted prevention program for disruptive elementary school children	August, G. J.', 'Egan, E. A.', 'Realmuto, G. M.', 'Hektner, J. M.'	2003	Wrong outcomes
Patterns and Temporal Changes in Peer Affiliation among Aggressive and Nonaggressive Children Participating in a Summer School Program	Hektner, J. M.', 'August, G. J.', 'Realmuto, G. M.'	2000	Wrong study design
Physical activity in middle school-aged children participating in a school-based recreation program	Kien, C. L.', 'Chiodo, A. R.'	2003	Wrong outcomes
Pilot study: Effects of short-term summer school program on plasma cognitive marker and non-lipid cardiovascular risk factors in female adolescents	Choi, M. D.', 'Park, K. S.'	2017	Wrong literature type
Pilot testing of an intensive cooking course for New Zealand adolescents: The create-our-own kai study	Black, K.', 'Thomson, C.', 'Chryssidis, T.', 'Finigan, R.', 'Hann, C.', 'Jackson, R.', 'Robinson, C.', 'Toldi, O.', 'Skidmore, P.'	2018	Wrong outcomes
Preparation For Medical School via an Intensive Summer Program for Future Doctors: A Pilot Study of Student Confidence and Reasoning Skills	Musick, David W.', 'Ray, Richard H.'	2016	Wrong population
Preventing Summer Learning Loss: Results of a Summer Literacy Program for Students from Low-SES Homes	Bowers, Lisa M. Schwarz, Ilsa	2017	Wrong outcomes
Process evaluation of an up-scaled community based child obesity treatment program: NSW Go4Fun	Welsby, D.', 'Nguyen, B.', O'Hara, B. J., 'Innes-Hughes, C.', 'Bauman, A.', 'Hardy, L. L.'	2014	Wrong intervention
Process evaluation of an up-scaled community based child obesity treatment program: NSW Go4Fun R	Welsby, Debra', 'Nguyen, Binh', O'Hara, Blythe J., 'Innes-Hughes, Christine', 'Bauman, Adrian', 'Hardy, Louise L.'	2014	Wrong intervention
Program development of a community based therapeutic day camp for children classified as emotionally disturbed	Bailey, Melisa B.'	2010	Wrong literature type
Promoting health and activity in the summer trial: Implementation and outcomes of a pilot study	Evans, E. Whitney; Bond, Dale S.; Pierre, Denise F.; Howie, Whitney C.; Wing, Rena R.; Jelalian, Elissa	2018	Wrong outcomes

Promoting physical activity through walking to treat childhood obesity, mainly for mild to moderate obesity	Yoshinaga, M.', 'Miyazaki, A.', 'Aoki, M.', 'Ogata, H.', 'Ito, Y.', 'Hamajima, T.', 'Tokuda, M.', 'Lin, L.', 'Horigome, H.', 'Takahashi, H.', 'Nagashima, M.'	2020	Wrong intervention
Promotion of physical activity and adequate nutrition in children during the summer school holidays	Perez-Lizaur, A. B.', 'Melendez-Mier, G.', 'Rocha-Aguilar, R.', 'Haua-Navarro, K.', 'Perez-Rodriguez, M.', 'Pffefer, F.'	2011	Wrong literature type
Rebound Body Mass Index Growth in Year-Round Elementary Education Students of Largely Hispanic Descent Undergoing Obesity Interventions	Alexander, A. G.', 'Lyons, P. E.'	2016	Wrong intervention
S.P.L.A.S.H. into fitness! an identity-focused behavioral swim camp and family-oriented ehealth intervention for girls	Kramer, Eydie Noelle'	2020	Wrong literature type
School Supplies and Financial Literacy for Families in Poverty	Morris, Ronald V.', 'Shockley, Denise'	2022	Wrong literature type
School-based fitness changes are lost during the summer vacation	Carrel, Aaron L.', 'Clark, R. Randall', 'Peterson, Susan', 'Eickhoff, Jens', 'Allen, David B.'	2007	Wrong intervention
School-based mental health prevention activities for homeless and at-risk youth	Nabors, L.', 'Proescher, E.', 'DeSilva, M.'	2001	Wrong outcomes
School-Based Weight Management Program Curbs Summer Weight Gain Among Low-Income Hispanic Middle School Students	Reesor, L.', 'Moreno, J. P.', 'Johnston, C. A.', 'Hernandez, D. C.'	2019	Wrong intervention
School's out: What are urban children doing? the summer activity study of somerville youth (SASSY)	Tovar, A.', 'Lividini, K.', 'Economos, C. D.', 'Folta, S.', 'Goldberg, J.', 'Must, A.'	2010	Wrong study design
Seasonal differences in patient retention and BNI changes in pediatric weight management treatment	Pike, G.', 'Boyer, K.', 'LeQuia, L.', 'Stratbucker, W.', 'Cadieux, A.', 'Silver, L.', 'Tucker, J.'	2021	Wrong literature type

Seasonal variability in body mass index change among children enrolled in the Pediatric Obesity Weight Evaluation Registry: A step in the right direction	Lane, T. S.', 'Sonderegger, D. L.', 'Binns, H. J.', 'Kirk, S.', 'Christison, A. L.', 'Novick, M.', 'Tucker, J.', 'King, E.', 'Wallace, S.', 'Brazendale, K.', 'Kharofa, R. Y.', 'Walka, S.', 'Heer, H. D. D.'	2023	Wrong intervention
Self-monitoring during whole group reading instruction: Effects among students with emotional and behavioral disabilities during summer school intervention sessions	Rafferty, L. A.'	2012	Wrong outcomes
Staking out the Successful Student	Brown, Christopher'	2005	Wrong study design
STEMming the Swell of Absenteeism in the Middle Years: Impacts of an Urban District Summer Robotics Program	Mac Iver, Martha Abele', 'Mac Iver, Douglas J.'	2019	Wrong outcomes
Summer School Effects in a Randomized Field Trial	Zvoch, Keith', 'Stevens, Joseph J.'	2013	Wrong population
Supporting at-risk youth and their families to manage and prevent diabetes: Developing a national partnership of medical residency programs and high schools	Gefter, L.', 'Morioka-Douglas, N.', 'Srivastava, A.', 'Rodriguez, E.'	2016	Wrong study design
Testing the effect of summer camp on excess summer weight gain in youth from low-income households: a randomized controlled trial	Evans, E. W.', 'Wing, R. R.', 'Pierre, D. F.', 'Howie, W. C.', 'Brinker, M.', 'Jelalian, E.'	2020	Wrong outcomes
The diet of children attending a holiday programme in the uk: Adherence to uk food-based dietary guidelines and school food standards	Crilley, E.', 'Brownlee, I.', 'Defeyter, M. A.'	2022	Wrong intervention
The Effect of Teacher-Family Communication on Student Engagement: Evidence from a Randomized Field Experiment	Kraft, Matthew A.', 'Dougherty, Shaun M.'	2013	Wrong outcomes
The effects of a children's summer camp programme on weight loss, with a 10 month follow-up	Gately, Paul Cooke, Carlton Butterly, R. J. Mackreth, P. Carroll, Sean	2000	Wrong outcomes
The Effects of Summer Sports School Basketball Training on Respiratory Functions of Female Students	Orhan, Serdar', 'Eskiyecek, Canan GÃ¼lbin'	2018	Wrong outcomes

The energykids pilot study: Comparing energy balance of primary school children during school and summer camp	Franchini, C.', 'Rosi, A.', 'Ricci, C.', 'Scazzina, F.'	2021	Wrong outcomes
The EnergyKids project: Pilot study on the energy balance of primary school children during school days and summer camp days	Rosi, A.', 'Franchini, C.', 'Scazzina, F.'	2020	Wrong literature type
The Fun, Food, and Fitness Project (FFFP): the Baylor GEMS pilot study	Baranowski, Tom Baranowski, Janice Cullen, Karen W. Thompson, Deborah Nicklas, Theresa A. Zakeri, Issa Rochon, James	2003	Wrong study design
The girls creating games program: An innovative approach to integrating technology into middle school	Denner, J.'	2007	Wrong outcomes
The Immediate and Lasting Effects of Resident Summer Camp on Movement Behaviors Among Children	Kidokoro, Tetsuhiro', 'Minatoya, Yuji', 'Imai, Natsuko', 'Shikano, Akiko', 'Noi, Shingo'	2022	Wrong outcomes
The impact of free access to swimming pools on children's participation in swimming. A comparative regression discontinuity study	Higgerson, J.', 'Halliday, E.', 'Ortiz-Nunez, A.', 'Barr, B.'	2019	Wrong outcomes
The impact of summer programming on the obesogenic behaviors of children: Behavioral outcomes from a quasi-experimental pilot trial	Dugger, R.', 'Brazendale, K.', 'Hunt, E. T.', 'Moore, J. B.', 'Turner-Mcgrievy, G.', 'Vogler, K.', 'Beets, M. W.', 'Armstrong, B.', 'Weaver, R. G.'	2020	Wrong outcomes
The impact of summer vacation on children's obesogenic behaviors and body mass index: a natural experiment	Weaver, R. G.', 'Armstrong, B.', 'Hunt, E.', 'Beets, M. W.', 'Brazendale, K.', 'Dugger, R.', 'Turner-McGrievy, G.', 'Pate, R. R.', 'Maydeu-Olivares, A.', 'Saelens, B.', 'Youngstedt, S. D.'	2020	Wrong outcomes
The programme 'Smoking and Me' has exerted its impact on elementary schoolchildren already for three years	Hruba, D.', 'Kachlik, P.'	2000	Wrong study design
Thinking outside the meals: How a community collaborative summer meals program influenced nutrition knowledge and skills, physical activity, and social interaction among Mexican-origin children	Sharkey, J.', 'Valdez, E.', 'Beltran, E.', 'Beltran, D.', 'Bustillos, B.'	2014	Wrong literature type

Training, muscle volume, and energy expenditure in nonobese American girls	Eliakim, A.', 'Scheett, T.', 'Allmendinger, N.', 'Brasel, J. A.', 'Cooper, D. M.'	2001	Wrong outcomes
Transformative Performing Arts and Mentorship Pedagogy: Nurturing Developmental Relationships in a Multidisciplinary Dance Theatre Program for Youth	Kane, Kevin M.'	2014	Wrong study design
Treating childhood obesity by walking: A randomised controlled trial	Yoshinaga, M.', 'Seki, S.', 'Ogata, H.', 'Ito, Y.', 'Aoki, M.', 'Miyazaki, A.', 'Tokuda, M.', 'Lin, L.', 'Horigome, H.', 'Nagashima, M.'	2017	Wrong intervention
Turn up the healthy eating and activity time (HEAT): Physical activity outcomes from a 4-year non-randomized controlled trial in summer day camps	Brazendale, Keith; Beets, Michael W.; Weaver, R. Glenn; Turner-McGrievy, Gabrielle; Moore, Justin B.; Huberty, Jennifer; Ward, Dianne S.	2020	Wrong outcomes
Understanding physical activity patterns among rural Aboriginal and non-Aboriginal young people	Macniven, R.', 'Richards, J.', 'Turner, N.', 'Blunden, S.', 'Bauman, A.', 'Wiggers, J.', 'Gwynn, J.'	2019	Wrong study design
University-School Partnerships: On the Impact on Students of Summer Schools (for School Students Aged 17-18) Run by Bristol ChemLabs	Shaw, A. J.', 'Harrison, T. G.', 'Croker, S. J.', 'Medley, M.', 'Sellou, L.', 'Shallcross, K. L.', 'Williams, S. J.', 'Grayson, D. J.', 'Shallcross, D. E.'	2010	Wrong population
Use of the School Setting During the Summer Holidays: Mixed-Methods Evaluation of Food and Fun Clubs in Wales	Morgan, Kelly', 'McConnon, Linda', 'Van Godwin, Jordan', 'Hawkins, Jemma', 'Bond, Amy', 'Fletcher, Adam'	2019	Wrong study design
Video game intervention for sexual risk reduction in minority adolescents: randomized controlled trial	Fiellin, L. E.', 'Hieftje, K. D.', 'Pendergrass, T. M.', 'Kyriakides, T. C.', 'Duncan, L. R.', 'Dziura, J. D.', 'Sawyer, B. G.', 'Mayes, L.', 'Crusto, C. A.', 'Forsyth, B. W. C.', 'Fiellin, D. A.'	2017	Wrong intervention

Ya gotta have friends: Social support and self-efficacy predict success following immersion treatment	Sampat, Sonia Kirschenbaum, Daniel S. Gierut, Kristen J. Germann, Julie N. Krawczyk, Ross	2014	Wrong study design
Youth empowerment solutions for violence prevention	Reischl, T. M.', 'Zimmerman, M. A.', 'Morrel-Samuels, S.', 'Franzen, S. P.', 'Faulk, M.', 'Eisman, A. B.', 'Roberts, E.'	2011	Wrong literature type
