# Supplementary material for: Effect of Summer Holiday Programs on Children’s Mental Health and Well-Being: Systematic Review and Meta-Analysis
Source: Children (Basel). 2024 Jul 23;11(8):887. doi: 10.3390/children11080887 (PMC11352663; doi:10.3390/children11080887)
Supplement: Supplementary file 1 [file children-11-00887-s001.zip › File S5. Thematic Coding of Study and Program Characteristics.pdf]

Supplementary File S5. Thematic Coding of Program Characteristics.

Explanation of Thematic Coding

**Item**	**Explanation**	**Code**
Participant characteristics		
Age	1. Based on mean age of participants: Year/Grade levels:<br>• R/K-2 (5-7 yrs): Lower primary<br>• G3-5 (8-10 yrs): Mid primary<br>• G6-8 (11-13 yrs): Upper primary<br>• G9+ (14+ yrs): High school<br>2. Mixed: More than one age group<br>3. If mean NR, based on the age range of 75% of participants<br>4. If more than one category = mixed	• Lower primary<br>• Mid primary<br>• Upper primary<br>• High school<br>• Mixed
SES	SES of the population<br>• Low: Low SES, "underprivileged"<br>• High: High SES, "privileged"<br>• Mixed: more than one SES band	• Low<br>• High<br>• Mixed
Program characteristics: Structure		
Type	Residential, day or home program:<br>• Residential includes accommodation with overnight stays<br>• Day programs are conducted outside of the home with children returning home at the end of every day<br>• Home programs are conducted at home, with/without an initial introductory/measurement session.<br>• Mixed programs are conducted in more than one setting	• Residential<br>• Day<br>• Home<br>• Mixed
Setting	Place the program is delivered:<br>• Community: including public spaces, community organisations<br>• School: educational facilities including schools, boarding schools, summer schools or universities<br>• Home	• Community<br>• School<br>• Home
Content	Specific program or general<br>• Specific (curriculum designed to address specific skill or behaviour)<br>• General (enrichment – program centred around games, toys, play or recreation)	• Specific<br>• General

Cost	Cost to participants (program funding):<br>• Free: no charge to participants e.g., program already established and externally funded<br>• Funded: no charge to participants as the program was funded for a brief period e.g., trial funding<br>• Paid: Participants paid to attend. Full or partial scholarships may have been available to some participants.	• Free<br>• Funded<br>• Paid
Program characteristic: Contact		
Duration	Time between first and last program session. Where programs had options for attendance (e.g., four weeks or eight weeks, these are stated as a range)	Number of weeks
Daily contact time	Contact hours per day:<br>• Sessional (2 hours or less per visit)<br>• Half day (>2 & <6 hours)<br>• School day (6-7 hours)<br>• Full day (>7 hours) day	• Sessional<br>• Half day<br>• School<br>• Full day
Intensity	Average Number of face-to-face contact sessions with the program per week. Calculated by dividing the number of sessions by the total weeks (start to finish). Occasional programs may have had one workshop session per month or two formal sessions throughout summer combined with homework.	• Daily (4-5 x week)<br>• Bi-weekly (2x week),<br>• Occasional (1 or less per week)
Other features	Factors that were reported and that supported attendance. Extra enrichment activities listed. Specifically, if meals (M) and transport to and from the program (T) were reported.	• M: Meals<br>• T: Transport
Attendance	Attendance level recorded and reported as % of scheduled sessions.<br>• 50% (low)<br>• 51-65% (moderate)<br>• 66-100% (high)	• Low<br>• Moderate<br>• High

Key: G: Grade. SES: Socioeconomic Status
