# Supplementary material for: Effect of Summer Holiday Programs on Children’s Mental Health and Well-Being: Systematic Review and Meta-Analysis
Source: Children (Basel). 2024 Jul 23;11(8):887. doi: 10.3390/children11080887 (PMC11352663; doi:10.3390/children11080887)
Supplement: Supplementary file 1 [file children-11-00887-s001.zip › File S6. Mental Health Meta analysis with sensitivity.pdf]

Supplementary File S6. Meta Analysis Summary of Results with Sensitivity Analysis.

**Analysis**	***SMD***	**95% CI**	***p***	**95%PI**	**I^2**	**95%CI**
**Mental Health**						
Anxiety & Depression						
Main analysis	-0.170	-2.935, 2.595	0.578	n/a	13%	n/a
Influential Cases Removed	n/a					
High Risk of Bias Removed	n/a (one study remains)					
Fixed effects	-0.159					
Psychological distress						
Main analysis	-0.458	-1.708, 0.791	0.255	-6.395, 5.479	50.9%	0%, 86%
Influential Cases Removed	-0.184	-2.641, 2.272	0.515	n/a	0%	n/a
High Risk of Bias Removed	n/a (one study remains)					
Fixed effects meta-analysis	-0.410					
**Self-perception**						
Self-esteem						
Main analysis	0.021	-0.017, 0.059	0.209	-0.053, 0.095	0%	0%, 75%
Influential Cases Removed	0.050	-0.016, 0.115	0.101	-0.101, 0.201	0%	0%, 79%
High Risk of Bias Removed	n/a	(all high RoB)				
Fixed effects meta-analysis	0.021					
Self-worth						
Main analysis	0.055	0.002, 0.108	0.047	-1.107, 1.216	0%	0%, 90%
Influential Cases Removed	0.050	-0.015, 0.115	0.101	-0.101, 0.201	0%	0%, 79%
High Risk of Bias Removed	n/a	(all high RoB)				
Fixed effects meta-analysis	0.055					

Sensitivity Analyses – Influential cases omitted

**Outcome**	**Influential cases**	**High Risk of Bias studies**
Anxiety-depression	(n/a - 2 studies only)	
Psychological distress	Levy 2020	n/a (leaves only 1 study)
Self-esteem	Thurber 2006	n/a (all high RoB)
Self-worth	Nil	n/a (all high RoB)
