# Supplementary material for: Effect of Summer Holiday Programs on Children’s Mental Health and Well-Being: Systematic Review and Meta-Analysis
Source: Children (Basel). 2024 Jul 23;11(8):887. doi: 10.3390/children11080887 (PMC11352663; doi:10.3390/children11080887)
Supplement: Supplementary file 1 [file children-11-00887-s001.zip › File S7. Mental health subgroup analyses.pdf]

Supplementary File S7. Subgroup analyses.

Subgroup analyses were performed using count-based analysis based on participant and program characteristics as follows:

Participant Characteristics

1. Disadvantage
2. Age

Program Characteristics

1. Duration of program (1-3 weeks vs 3 weeks or longer)
2. Format (residential vs day program)
3. Content (specific mental, emotional or social wellbeing focus vs not)
4. Funding (paid in part/full by student vs no payment by student)
5. Daily contact (half day or less vs school day or full day)

Subgroup analysis

Outcomes

When studies reported multiple outcomes under the same broad construct (e.g., anxiety and depression under the broad concept of mental health), when findings were in the same direction, the strongest finding was retained. If findings were in conflicting directions of effect (e.g., one improved and one declined) a "mixed" finding was designated.

Strength of effect

Outcomes were coded based on the effect size and statistical significance as follows:

Effect	*Effect size (SMD, d, g)*	*Statistical significance (p-value)*
No change	0 to < 0.1	>0.05
Suggestive improvement	0.1 or >	>0.05
Significant improvement	0.1 or >	<0.05

Key

Each study is represented by an oval shape, with the study number in the centre. The study is coded according to findings as follows

25 Significant improvement

23 Suggestive improvement

9 No change

23 Mixed findings

23 Suggestive worsening

5 Significant worsening

Subgroup analysis 1: Disadvantage

Outcome	Disadvantaged groups	Not reported as disadvantaged
Mental health	13 23<br>12 16 21	5 6
Self-perception	3 18 23 24	1 8 10 26<br>9 11 20 22
Social-emotional wellbeing	3 19 23 4<br>15 21	2 5 9 10 26 7<br>1 22
Cognition	21	14 17
Other	23<br>16	20 22 25<br>9 5

Subgroup analysis 2: Age

Outcome	Primary school	Middle school	High school
Mental health	6<br>12	21	13<br>5 16
Self-perception	3 10 18<br>11 22	1 24 26<br>20	
Social-emotional wellbeing	2 3 10 11 7<br>22 15	19 26<br>1 21	5 4
Cognition		17 20 21	
Other	22	25	5

Studies of mixed age ranges not included: 1 8 9 14 19 23

Primary school children: kindergarten/reception to grade six (ages five to 11 years)
Middle school students: grades 7-9 (ages 12-14 years)
High-school students: grades 10-12 (ages 15 years and older)

Subgroup analysis 3: Format

Outcome	Residential programs	Day programs
Mental health	21	21 13 23<br>5 12 16
Self-perception	8 18 26<br>20	1 3 10 23 24<br>9 11
Social-emotional wellbeing	26<br>21	2 3 5 10 11 19 23<br>1 15
Cognition	21	
Other	20	9<br>5

Not including: home programs (k=2), or not-reported formats (k=6)

Subgroup analysis 4: Content

Outcome	Specific mental, emotional, social wellbeing curriculum	Other curriculum
Mental health	13 23<br>5 12 16	6 21 23
Self-perception	10 23 26	1 3 8 18 23 24<br>11 20 22
Social-emotional wellbeing	2 5 10 23 26 4 7<br>15	3 11 19 23<br>1 22 21
Cognition		14 17 21
Other	25<br>5	22 23<br>20

Not including: not specified (k=3).
Note: Travis 2019 had a specific mental health group and a general group.

Subgroup 5: Daily contact

Outcome	Shorter contact: Half day or less	Longer contact: School day or full day
Mental health	5	6 13 23<br>12 16 21
Self-perception	1 10 24	3 8 18 23 26<br>11 20
Social-emotional wellbeing	1 5 9 7	2 3 11 19 23 26<br>15 21
Cognition		17 21
Other	25<br>5	20

Subgroup 6: Program duration

Outcome	Short duration: 1-3 weeks	Longer duration: 3 weeks or more
Mental health	6<br>5 21	23<br>12 16
Self-perception	10 18 26<br>20	1 2 23 24<br>11
Social-emotional wellbeing	5 10 19 26 4<br>21	2 3 11 23<br>1 15
Cognition	14 17 21	
Other	20 5	

Not including studies that had a range of attendance (e.g., 2-6 weeks, k=7) or non-specified duration (k=5).

Subgroup 7: Funding

Outcome	Funding: paid in part or full by student	No payment from student
Mental health		5 12 16 21
Self-perception	8 18 26<br>11 20	24<br>22
Social-emotional wellbeing	11 26	19 2 5 4<br>22 15 21
Cognition		21
Other	20	22 25<br>16 5

Funding for participation was not reported in k=10 studies

List of included studies[1-26]

1. Anderson-Butcher D, Iachini AL, Riley A, Wade-Mdivanian R, Davis J, Amorose AJ. Exploring the impact of a summer sport-based youth development program. *Evaluation and program planning*. 2013;37(NA):64-69. doi:10.1016/j.evalprogplan.2013.01.002
2. Ay SC, Keskin HK, Akilli M. Examining the effects of negotiation and peer mediation on students' conflict resolution and problem-solving skills. Article. *International Journal of Instruction*. 2019;12(3):717-730. doi:10.29333/iji.2019.12343a
3. Bethea SL. The Impact of Oakland Freedom School's Summer Youth Program on the Psychosocial Development of African American Youth. Article. *Journal of Black Psychology*. 2012;38(4):442-454. doi:10.1177/0095798411431982
4. de los Pinos CC, Soto AG, Conty JLM, Serrano RC. Summer Camp: Enhancing Empathy Through Positive Behavior and Social and Emotional Learning. *Journal of Experiential Education*. 2020;43(4):105382592092338-415. doi:10.1177/1053825920923382
5. Exner-Cortens D, Wolfe D, Crooks CV, Chiodo D. A preliminary randomized controlled evaluation of a universal healthy relationships promotion program for youth. *Canadian Journal of School Psychology*. 2020;35(1):3-22. doi:https://dx.doi.org/10.1177/0829573518821508
6. Fainardi V, Fasola S, Mastrorilli C, Volta E, La Grutta S, Vanelli M. A two-week summer program promoting physical activity: quality of life assessment in Italian children. *Psychology, health & medicine*. 2020;26(4):444-456. doi:10.1080/13548506.2020.1761552
7. Fujieda S. A practical study of the social skills training at home for elementary school children during summer and winter vacation. Empirical Study; Quantitative Study. *Japanese Journal of Counseling Science*. Dec 2011;44(4):313-322.
8. Gately P, Cooke C, Barth JH, Bewick BM, Radley D, Hill AJ. Children's Residential Weight-Loss Programs Can Work: A Prospective Cohort Study of Short-Term Outcomes for Overweight and Obese Children. *Pediatrics*. 2005;116(1):73-77. doi:10.1542/peds.2004-0397
9. Gerber Y, Gentaz E, Malsert J. The effects of Swiss summer camp on the development of socio-emotional abilities in children. *PloS one*. 2022;17(10):e0276665-e0276665. doi:10.1371/journal.pone.0276665
10. Goodyear M, Cuff R, Maybery D, Reupert A. CHAMPS: A peer support program for children of parents with a mental illness. Empirical Study; Quantitative Study. *AeJAMH (Australian e-Journal for the Advancement of Mental Health)*. Dec 2009;8(3):296-304. doi:https://dx.doi.org/10.5172/jamh.8.3.296
11. Henert S, Jacobs J, Wahl-Alexander Z. Let's play! Exploring the impact of summer day camp participation on the physical and psychosocial experiences of diverse urban youth. Review. *Child & Adolescent Social Work Journal*. May 2021;38(4):381-391. doi:https://dx.doi.org/10.1007/s10560-021-00769-6
12. Hopkins LC, Holloman C, Melnyk B, et al. Participation in structured programming may prevent unhealthy weight gain during the summer in school-aged children from low-income neighbourhoods: feasibility, fidelity and preliminary efficacy findings from the Camp NERF study. *Public Health Nutrition*. 2019;22(6):1100-1112. doi:10.1017/S1368980018003403
13. Levy I, Travis R. The Critical Cycle of Mixtape Creation: Reducing Stress via Three Different Group Counseling Styles. *The Journal for Specialists in Group Work*. 2020;45(4):307-330. doi:10.1080/01933922.2020.1826614
14. Manjunath NK, Telles S. Spatial and verbal memory test scores following yoga and fine arts camps for school children. *Indian journal of physiology and pharmacology*. 2004;48(3):353-356. doi:NA
15. Nabors L, Proescher E, DeSilva M. School-based mental health prevention activities for homeless and at-risk youth. Article. *Child and Youth Care Forum*. 2001;30(1):3-18. doi:10.1023/A:1016634702458

16. Pierce BA, Bowden B, McCullagh M, et al. A Summer Health Program for African-American High School Students in Baltimore, Maryland: Community Partnership for Integrative Health. *Explore (New York, NY)*. 2017;13(3):186-197. doi:10.1016/j.explore.2017.02.002
17. Pradhan B, Nagendra HR. Effect of yoga relaxation techniques on performance of digit-letter substitution task by teenagers. *International Journal of Yoga*. 2009;2(1):30-4.
18. Readdick CA, Schaller GR. Summer camp and self-esteem of school-age inner-city children. *Perceptual and motor skills*. 2005;101(1):121-130. doi:10.2466/pms.101.1.121-130
19. Riley A, Anderson-Butcher D, Logan JAR, Newman TJ, Davis J. Staff Practices and Social Skill Outcomes in a Sport-Based Youth Program. *Journal of Applied Sport Psychology*. 2016;29(1):59-74. doi:10.1080/10413200.2016.1179700
20. Saunders-Ferguson K, Barnett RV, Culen G, Tenbroeck S. Self-esteem assessment of adolescents involved in horsemanship activities. Article. *Journal of Extension*. 2008;46(2)2fea6.
21. Smith BH, Kim H, Esat G, et al. Comparing Three Overnight Summer Camp Experiences for Marginalized Middle School Students: Negative, Neutral, and Positive Results. *Journal of Experiential Education*. 2022;45(2):136-156. doi:https://doi.org/10.1177/10538259211030529
22. Thurber CA, Scanlin MM, Scheuler L, Henderson KA. Youth Development Outcomes of the Camp Experience: Evidence for Multidimensional Growth. *Journal of youth and adolescence*. 2006;36(3):241-254. doi:10.1007/s10964-006-9142-6
23. Travis R, Jr., Gann E, Crooke AH, Jenkins SM. Hip hop, empowerment, and therapeutic beat-making: Potential solutions for summer learning loss, depression, and anxiety in youth. Empirical Study; Quantitative Study. *Journal of Human Behavior in the Social Environment*. Aug-Sep 2019;29(6):744-765. doi:https://dx.doi.org/10.1080/10911359.2019.1607646
24. Ullrich-French S, McDonough MH, Smith AL. Social Connection and Psychological Outcomes in a Physical Activity-Based Youth Development Setting. *Research quarterly for exercise and sport*. 2012;83(3):431-441. doi:10.1080/02701367.2012.10599878
25. Werch CE, Carlson JM, Pappas DM, Edgemon P, DiClemente CC. Effects of a brief alcohol preventive intervention for youth attending school sports physical examinations. *Substance Use and Misuse*. 2000;35(3):421-432. doi:10.3109/10826080009147704
26. Wong WW, Abrams SH, Mikhail C, et al. An innovative summer camp program improves weight and self-esteem in obese children. *Journal of pediatric gastroenterology and nutrition*. 2009;49(4):493-497. doi:10.1097/mpg.0b013e31819b5da2
