# Supplementary figures and images for: Effect of Summer Holiday Programs on Children’s Mental Health and Well-Being: Systematic Review and Meta-Analysis
Source: Children (Basel). 2024 Jul 23;11(8):887. doi: 10.3390/children11080887 (PMC11352663; doi:10.3390/children11080887)

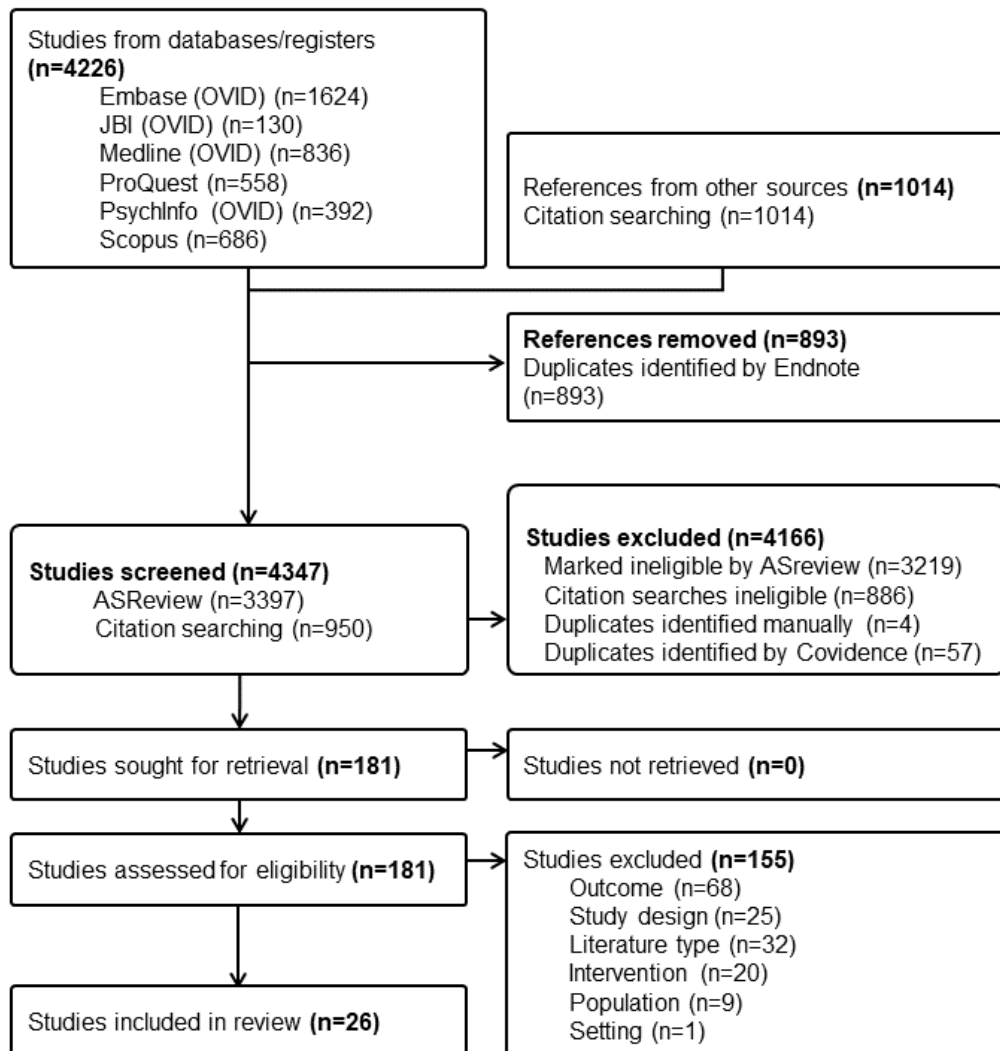

**Figure S1.** PRISMA flow diagram.

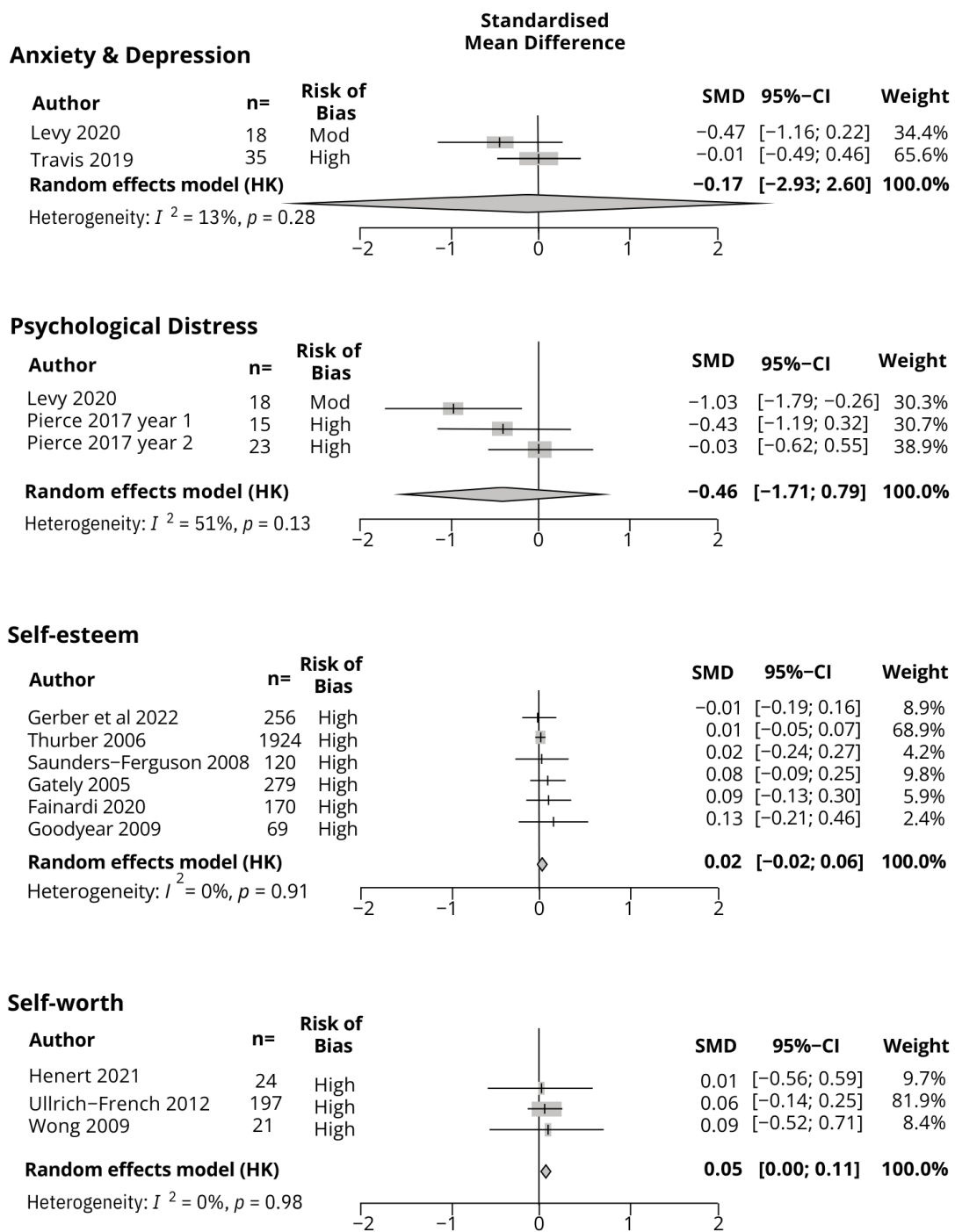

Figure S2. Mental health summer programs forest.

Supplement: Supplementary file 1 [file children-11-00887-s001.zip › Supplementary Figures.pdf]
